# Classification of primary angle closure spectrum with hierarchical cluster analysis

**DOI:** 10.1371/journal.pone.0199157

**Published:** 2018-07-23

**Authors:** Sasan Moghimi, Ali Torkashvand, Massood Mohammadi, Mehdi Yaseri, Luke J. Saunders, Shan C. Lin, Robert N. Weinreb

**Affiliations:** 1 Hamilton Glaucoma Center, Department of Ophthalmology, Shiley Eye Institute, University of California-San Diego, La Jolla, California, United States of America; 2 Department of Ophthalmology, Shiley Eye Institute, University of California, San Diego, La Jolla, California, United States of America; 3 Tehran University Research Center, Tehran University of Medical Science, Tehran, Iran; 4 Department of Epidemiology and Biostatistics, Tehran University of Medical Sciences, Tehran, Iran; 5 Koret Vision Center, University of California, San Francisco Medical School, San Francisco, California, United States of America; Bascom Palmer Eye Institute, UNITED STATES

## Abstract

**Purpose:**

To classify subjects with primary angle closure into clusters based on features from anterior segment optical coherence tomography (ASOCT) imaging and to explore how these clusters correspond to disease subtypes, including primary angle closure suspect (PACS), primary angle closure glaucoma(PACG), acute primary angle closure (APAC) and fellow eyes of APAC and reveal the factors that become more predominant in each subtype of angle closure.

**Method:**

A cross-sectional study of 248 eyes of 198 subjects(88 PACS eyes, 53 PACG eyes, 54 APAC eyes and 53 fellow eyes of APAC) that underwent complete examination including gonioscopy, A-scan biometry, and ASOCT. An agglomerative hierarchical clustering method was used to classify eyes based on ASOCT parameters.

**Results:**

Statistical clustering analysis produced three clusters among which the anterior segment parameters were significantly different. Cluster 1(43 eyes) had the smallest anterior chamber depth(ACD) and area, as well as the greatest lens vault (p<0.001 for all). Cluster 2(113 eyes) had the thickest iris at 2000 microns(p = 0.048), and largest iris area(p<0.001), and the deepest ACD (p<0.001). Cluster 3(92 eyes) was characterized by elements of both clusters 1 and 2 and a higher iris curvature(p<0.001). There was a statistically significant difference in the distribution of clusters among subtypes of angle closure eyes(p<0.001). Although the patterns of clusters were similar in PACS and PACG eyes, with the majority of the eyes classified into cluster 2(55%, and 62%, respectively), the highest proportion of APAC and fellow eyes were assigned to clusters 1(44%) and 3 (51%), respectively.

**Conclusion:**

Hierarchical cluster analysis identified three clusters with different features. Predominant anatomical components are different among subtypes of primary angle closure.

## Introduction

Primary angle closure glaucoma (PACG) is a leading cause of blindness in Asia. It is more visually damaging than primary open angle glaucoma.[1] In PACG, degenerative changes in the trabecular meshwork resulting from iridiotrabecular contact (ITC) lead to high intraocular pressure (IOP) resulting in glaucomatous optic atrophy.[2, 3]

Pupillary block is known as the principal mechanism in the pathogenesis of angle closure. Laser peripheral iridotomy (LPI), which eliminates pupillary block, is the standard treatment for primary angle closure (PAC).[4] However, LPI may not always be the best treatment for all subtypes of angle closure.[5, 6] About one fifth of PAC and PACG eyes continue to have residual angle closure in the presence of a patent LPI[3, 7] and thus other mechanisms such as iris configuration and forward movement of lens are considered to have important roles in the angle closure. Identifying these mechanisms for each patient could lead to better management of the disease by individualizing treatment.[8–10]

The subtypes of primary angle closure and their definitions have been shown in Table 1. We previously evaluated anterior segment optical coherence tomography (ASOCT) images qualitatively and demonstrated that there is a significant difference in underlying primary angle closure mechanisms among different subtypes of angle closure.[11, 12] Although pupillary block was the main responsible mechanism in fellow eyes of acute primary angle closure (APAC) and primary angle closure suspect (PACS) eyes, exaggerated lens vault was the responsible mechanism in about one half of the APAC eyes.

**Table 1:** Subtypes of angle closure and their definition in the present study.

Subtypes	Definition
PACS	Subjects with narrow angles (defined as eyes in which there was at least 270° of the posterior pigmented trabecular meshwork), **and** IOP ≤21mmHg, **and** normal optic disc and no PAS
PACG	Subjects with narrow angles (defined as above), **and** chronically elevated IOP above 21 mmHg, **and** glaucomatous optic neuropathy (diffuse or localized rim thinning, disc hemorrhage, a notch in the rim, or a vertical cup-to-disc ratio higher than the other eye by >0.2) and typical visual field defects (PSD with P <0 .05, and abnormal glaucoma hemifield test result)
APAC	Subjects with narrow angles (defined as above), **and** IOP at presentation of at least 30 mmHg, **and** presence of any two of the following symptoms: ocular or peri-ocular pain, nausea and/or vomiting, halos, **and** presence of at least 3 of the following examination findings: conjunctival injection, microcytic corneal edema, mid-dilated pupil, and shallow anterior chamber

APAC: Acute primary angle closure; IOP: Intraocular pressure; PACG: Primary angle closure glaucoma; PACS: Primary angle closure suspect; peripheral anterior synechiae: PAS; pattern standard deviation: PSD

Although this classification scheme may be effective for evaluation of the underlying mechanisms of PAC eyes, disagreement between observers is possible using this approach.[10, 12] Moreover, it is not easy to assign just one mechanism to some eyes. In another study, for example, more than one mechanism was reported in 35 out of 116 primary angle closure eyes.[12]

Recently, it was shown that PACS or PACG eyes could be grouped based on ASOCT imaging and hierarchical clustering analysis into two or three different clusters.[8, 13] One cluster has been characterized by a thick peripheral iris, a second cluster by a shallow anterior segment and large lens vault (LV), and the third by a mixture of these elements. In another study, Han et al[9] investigated the effect of LPI in clusters of angle closure eyes based on ASOCT-derived parameters and showed that the outcomes of LPI differed between clusters with specific anatomical characteristics.

To the best of our knowledge, no study has classified different angle closure subtypes using cluster analysis. The purpose of this study was to identify clusters in eyes with angle closure and explore their distribution in different subtypes. We hypothesize that hierarchical cluster analysis will help reveal the factors that become more predominant in each subtype of angle closure and their appropriate management.

## Method

The Farabi Angle Closure Study (FACS) protocol followed the tenets of the Declaration of Helsinki and was approved by the Tehran University of Medical Sciences Ethics Committee. In this prospective observational comparative study, patients with primary angle closure were recruited consecutively from the glaucoma service at Farabi Eye Hospital between November 2010 to November 2016. All participants gave written informed consent and were classified into one of the following four subtypes: Primary angle closure suspects (PACS), Primary angle closure glaucoma (PACG), acute primary angle closure (APAC), and *Fellow* eyes of APAC. (Table 1). *Fellow* eyes of APAC were eyes with no history or signs of prior acute glaucoma attack.

Attacks were broken in APAC eyes with oral acetazolamide, intravenous mannitol or oral glycerin, topical timolol, and topical brimonidine. When IOP was less than 21 mmHg (with or without medication) and when signs and symptoms of acute IOP rise had subsided, the APAC attack was considered broken. Eyes whose attack could not be broken with these medications were excluded from the study and received appropriate intervention. In PACS and PACG patients, only the right eyes of patients were included for analysis in this study. If the left eye was the only affected eye, the left eye was included.

### Examinations

Study eyes underwent a complete eye examination, which included best-corrected visual acuity, refraction, corneal pachymetry, slit lamp examination, intraocular pressure measurement with applanation tonometry, gonioscopy, fundus examination, biometry (IOLMaster; Carl Zeiss Meditec, San Leandro, CA, USA), and achromatic visual field testing (program 24–2, Swedish Interactive Threshold Algorithm standard, model 750, Humphrey Field Analyzer, Carl Zeiss Meditec, Dublin, CA, USA). Gonioscopy was performed in a dark room by a glaucoma specialist (SM) using a Zeiss-style four-mirror goniolens (Model G-4, Volk Optical, Mentor, OH, USA). The Shaffer grading system was used to evaluate the angle on gonioscopy. Number of medications used in each eye were documented.

### Anterior segment optical coherence tomography

**Fig 1:**
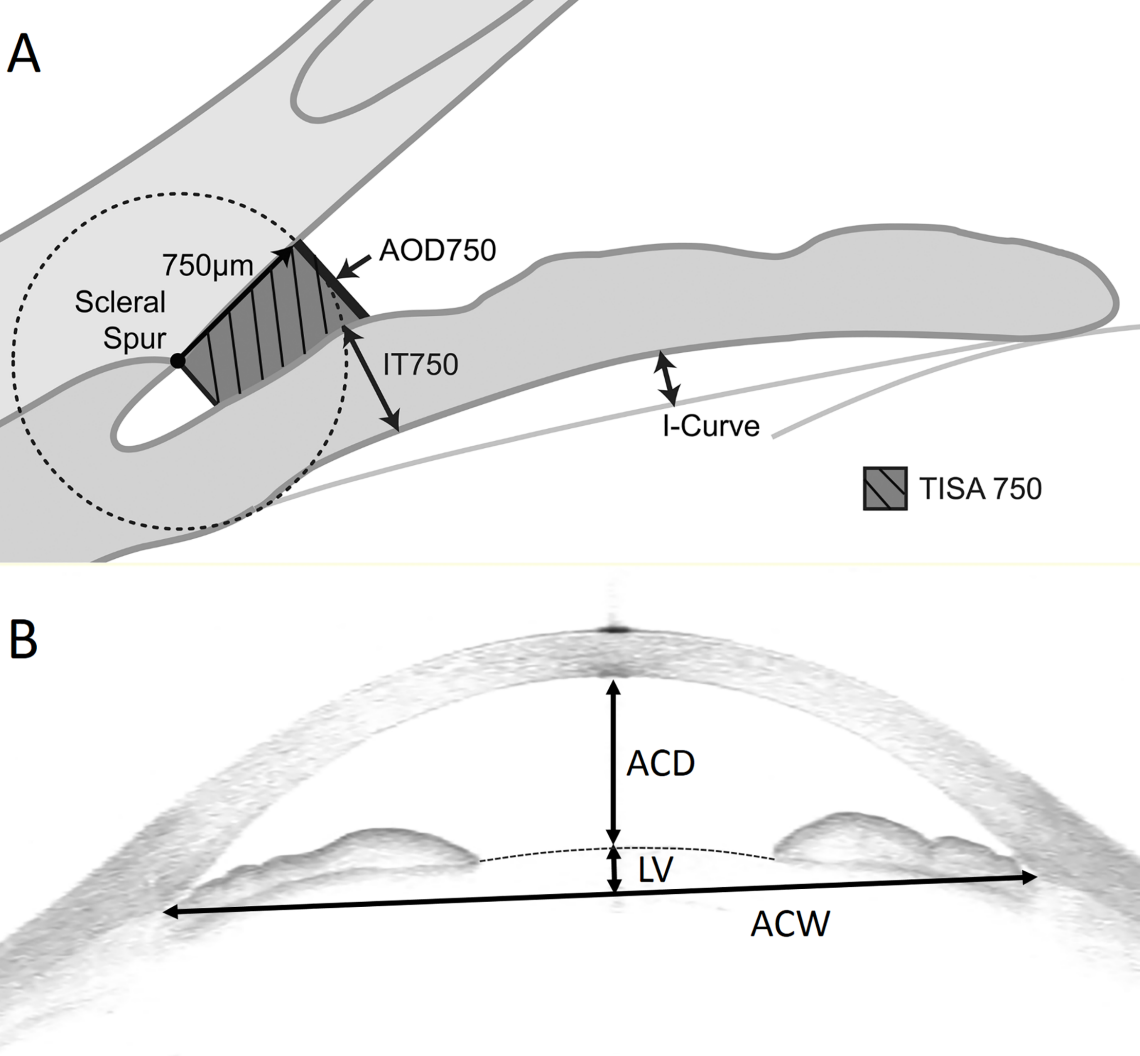
A and B. ASOCT image showing angle opening distance and trabecular iris surface area at 750 microns from the scleral spur (AOD750, TISA750), iris thickness (IT750), iris curvature (I-Curve), anterior chamber depth (ACD), anterior chamber width (ACW), and lens vault (LV).

Anterior segment optical coherence tomography (Visante, Carl Zeiss Meditec, Dublin, CA) examinations were done before laser peripheral iridotomy (LPI). This ASOCT system uses a long wavelength (1310 nm) to penetrate through tissues that highly scatter light such as sclera and limbus, allowing for visualization of the iridocorneal angle and entire cross section of anterior segments with a resolution of 10 to 18 mm. All ASOCT images were taken under the same dark conditions with the subject seated. In APAC eyes, the ASOCT exam carried out when the attack was broken with intensive therapy. Miotic or mydriatic medications were not used in any of the patients prior to imaging. Scans were centered on the undilated pupil, and were obtained along horizontal and vertical axes using the enhanced anterior segment single protocol (version 2.0). The same examiner, who was masked to clinical findings, obtained all images. Two images were captured for each axis, and the one with the higher quality and visibility of scleral spur was chosen for analysis using the Zhongshan Angle Assessment Program (ZAAP; Zhongshan Ophthalmic Center, Guangzhou, China). The algorithm then automatically calculated the anterior chamber and angle parameters. The following parameters were measured: anterior chamber depth (ACD), anterior chamber area (ACA), iris thickness at 750 microns (IT750), iris thickness at 2000 microns (IT2000), maximum central iris thickness (ITCM), iris area (I-Area), iris curvature (I-Curve), lens vault (LV), and various angle parameters including angle opening distance at 500 and 750 microns from the scleral spur (AOD500, AOD750) and trabecular iris surface area at 500 and 750 microns from the scleral spur (TISA500, TISA750).[2, 14](Fig 1) Lens vault (LV), defined as the perpendicular distance between the anterior lens pole and the horizontal line joining the temporal and nasal scleral spurs, is one of the novel parameters that can be measured with AS-OCT and has been associated with angle closure.[14, 15]

### Statistical analysis

Statistical analysis was performed using SPSS software version 22 (SPSS, Inc., Chicago, IL, USA). Parametric variables were analyzed using analysis of variance (ANOVA) and Bonferroni adjustments. Kruskal-Wallis was used for analysis of nonparametric variables. Analysis of qualitative variables was performed by chi-square test.

A two-step agglomerative hierarchical cluster analysis was used to classify primary angle closure eyes into distinct clusters according to ASOCT features.[16] This algorithm assigns the subject observations into clusters, using Schwarz’s Bayesian Information Criterion (BIC).[17] In this method of segmenting, a group of patients into clusters such that those within each cluster are more closely related to one another than those assigned to different clusters. This method is unbiased as no previous set standards are used for clustering. Compared to classical methods of cluster analysis, the two-step can handle quantitative and qualitative variables simultaneously. The distance measure was based on log-likelihood. The ratio of the change in the BIC at each successive merging determines the number of clusters; if this change is less than 0 it determines that the number of clusters should not increase by more than 1, otherwise the minimum cluster that would lead to a change by less than 0.04 was selected. To validate the classification approach, we divided our cases randomly in two datasets (A and B) with similar proportions of subtypes in each. The model for the first dataset was run and then it was rerun to analyze the chronologically independent second dataset.

## Results

Two APAC eyes were excluded from study due to unbroken acute attack necessitating early cataract surgery. After excluding 11 images of 11 subjects due to poor image quality or indistinct scleral spur, a total of 248 eyes were enrolled in the study. 88 eyes were PACS; 53 eyes were PACG, 53 eyes were *fellow* eyes and 54 eyes had APAC. The demographic and clinical examination data of the four subtypes are shown in Table 2. The mean age was not significantly different in the four study groups (PACS: 60.4±8.9; PACG: 59.1±9.5; *fellow* eyes of APAC: 59.0±9.0; APAC: 61.4±9.4 years; p = 0.492). The proportion of females were significantly larger in the PACS, APAC, and the *fellow* eyes of APAC than in the PACG eyes (p < 0.001). The PACG group had higher IOP at imaging when compared with the other groups (p < 0.001). The number of medications were greater in the APAC eyes, compared to other groups. Axial length and ACD were significantly greater in PACG subtype than in the APAC, and their *fellow* eyes of APAC (p < 0.001).

**Table 2:** Comparison of clinical characteristics among different subtypes of angle closure.

	PACS (No = 88)	PACG (No = 53)	Fellow eyes of APAC (No = 53)	APAC (No = 54)	P-value^a^
Gender (F:M)	53:35	25:28	38:15	39:15	0.021
Age (Mean ± SD) (Years) [Range]	60.4±8.9 (39–81)	59.1±9.5 (37–86)	59.0±9.0 (45–88)	61.4±9.4 (45–88)	0.492
IOP (Mean ± SD) (mmHg) [Range]	15.±2.8 (9–21)	19.9 ±7.9 (9–32)	12.5±3.3 (6–22)	14.5±5.4 (4–21)	<0.001 ^b^ ^c^ ^d^ ^e^ ^f^
No of medication (Mean ± SD) [Range]	0	1.2±1.3 (0–4)	0.8± 0.5 (0–2)	2.0 ±0.5 (0–4)	<0.001 ^b^ ^d^ ^c^^‖^^e^
Axial length (Mean ± SD) (mm) [Range]	22.12±0.83 (19.50±24.35)	22.58±0.84 (20.87±24.83)	21.75±1.09 (16.08±23.65)	21.83±1.13 (16.12±23.65)	<0.001 ^b^ ^e^

APAC: Acute primary angle closure; IOP: Intraocular pressure; PACG: Primary angle closure glaucoma; PACS: Primary angle closure suspect

^a^ Statistical significance tested by ANOVA.

Bonferroni post-hoc adjustment P<0.05 for

^b^ PACS vs PACG

^c^ PACS vs Fellow

^d^ PACS vs Fellow

^e^ PACG vs APAC

^f^ Fellow vs APAC.

The mean values of the anterior segment parameters in four subtypes are summarized in Table 3. APAC and their *fellow* eyes had the narrowest and the PACS group had the widest angle (ANOVA p <0.001, p<0.05 for pairwise comparison between PACS and the other groups) APAC group had the shallower ACD and ACA and greatest LV when compared with the rest of the subtypes but PACS and PACG eyes had comparable ACD, ACA, and LV. (ANOVA p < 0.001 for ACD, ACA, and LV, p<0.05 for all pairwise comparison except between PACS and PACG eyes). Although *fellow* eyes of APAC had the thickest IT750 (ANOVA p = 0.026, p<0.05 for pairwise comparison only between fellow eyes of APAC and PACS), IT2000 and ITCM, was similar among the subtypes. Iris curvature was found to be greater in the PACS and fellow eyes of APAC than PACG and APAC. (ANOVA p <0.001, p <0.05 for all pairwise comparison except between PACS and APAC eyes and PACG and fellow eyes of APAC).

**Table 3:** Comparison of angle and anterior segment parameters (Mean ± SD) measured by anterior segment optical coherence tomography in each subtype of angle closure.

	PACS (No = 88)	PACG (No = 53)	Fellow eye of APAC (No = 53)	APAC (No = 54)	P-value^a^
**Angle Parameters**					
AOD500 (mm)	0.104±0.075	0.064±0.059	0.029±0.037	0.008±0.025	<0.001^b^ ^c^ ^d^
AOD750 (mm)	0.151±0.104	0.135±0.081	0.062±0.056	0.053±0.053	<0.001^b^ ^c^ ^d^
TISA500 (mm^2^)	0.049±0.032	0.024±0.024	0.021±0.025	0.011±0.015	<0.001^b^ ^c^ ^d^
TISA750 (mm^2^)	0.087±0.051	0.054±0.039	0.037±0.033	0.023±0.022	<0.001^b^ ^c^ ^d^
**Anterior Chamber Parameters**					
ACD (mm)	2.13±0.25	2.12±0.28	1.96±0.21	1.84±0.24	<0.001 ^c^ ^d^ ^e^ ^f^
ACA (mm)	14.87±2.49	14.82±2.66	13.31±2.15	12.44±2.02	<0.001 ^c^ ^d^ ^e^ ^f^
ACW (mm)	11.31±0.48	11.33±0.48	11.25±0.51	11.22±0.48	0.596
Lens vault (μm)	813.9±255.7	802.1±250.2	968.8±185.5	1017.2±259.1	<0.001 ^c^ ^d^ ^e^ ^f^
**Iris Parameters**					
IT750 (mm)	0.47±0.10	0.45±0.08	0.50±0.09	0.48±0.09	0.026 ^d^
IT2000 (mm)	0.44±0.08	0.43±0.08	0.45±0.07	0.46±0.13	0.321
ITCM (mm)	0.60±0.08	0.58±0.07	0.61±0.07	0.61±0.14	0.281
I-Area (mm^2^)	1.53±0.25	1.52±0.27	1.53±0.23	1.49±0.26	0.772
I-Curve(mm)	0.37±0.29	0.29±0.12	0.36±0.14	0.30±0.11	<0.001 ^b^ ^d^ ^f^
Pupil diameter (mm)	3.91±0.96	3.89±0.85	4.02±0.83	4.26±0.80	0.080

APAC: Acute primary angle closure; ACD: Anterior chamber depth; ACW: Anterior chamber width; ACA: Anterior chamber area; AOD: Angle opening distance; CM: Central maximum; IT: Iris thickness; I-Area: Iris area; I-Curve: Iris curvature; PACG: Primary angle closure glaucoma; PACS: Primary angle closure suspect; TISA: Trabecular-iris surface area

^a^ Statistical significance tested by ANOVA.

Bonferroni post-hoc adjustment P<0.05 for

^b^ PACS vs PACG

^c^ PACS vs Fellow

^d^ PACS vs Fellow

^e^ PACG vs APAC

^f^ Fellow vs APAC.

**Fig 2:**
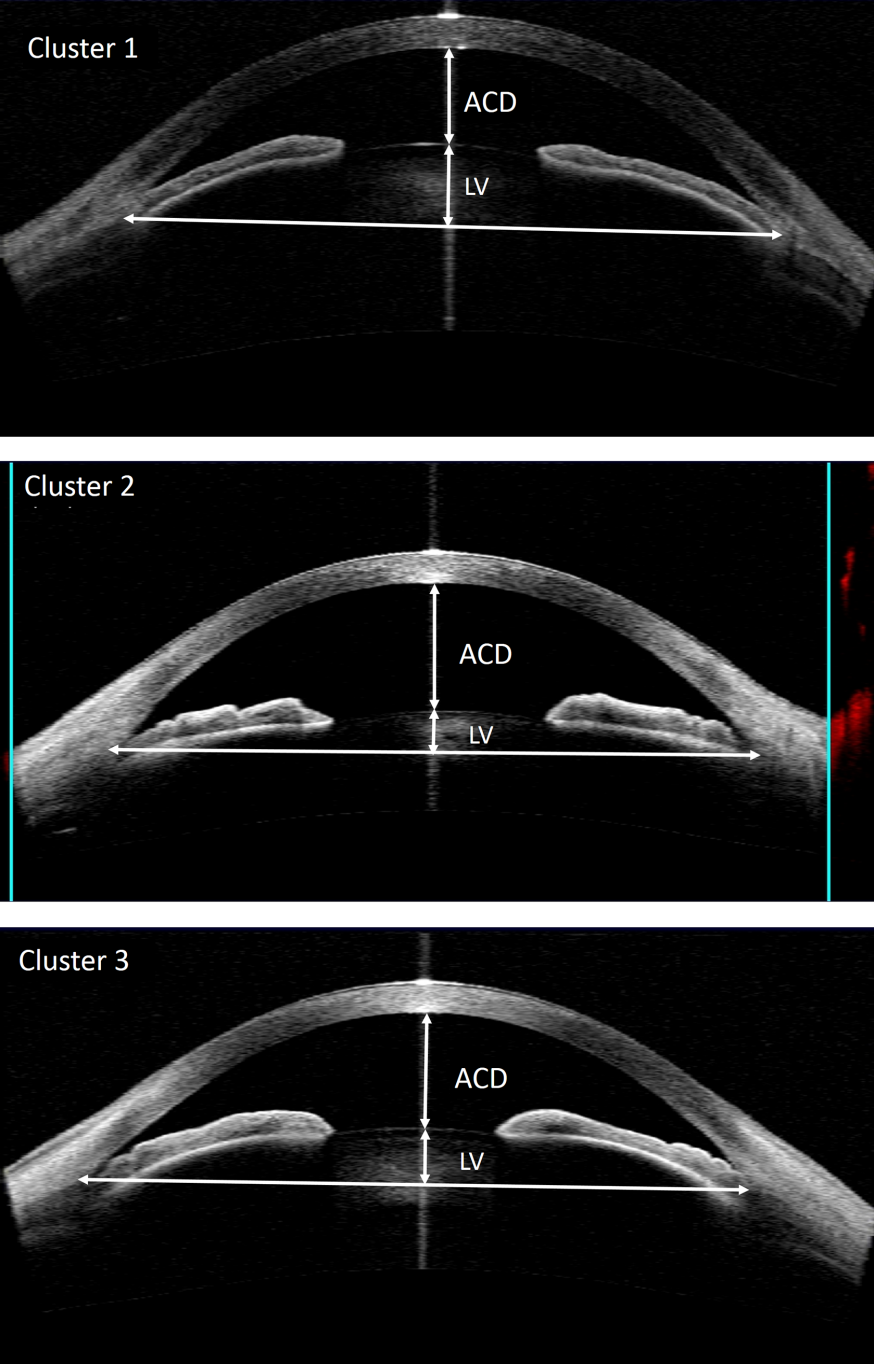
The anterior segment-optical coherence tomography (ASOCT) images of a typical case from each of the 3 clusters: Upper: A case from cluster 1 with shallow ACD and ACA and ACW, and a large lens vault: Middle: A case from cluster 2 with deep anterior chamber and thick iris, bottom: a case from cluster3 with intermediate ACD, ACA, LV, and high iris curvature.

The two-step cluster analysis produced three clusters, among which the anterior segment parameters were significantly different. The mean values of the anterior chamber parameters for each cluster are summarized in Table 4. The number of eyes that classified into cluster 2 was the greatest (113) followed by cluster 3 (92) and cluster 1 (43), respectively. Cluster 1 was characterized by a shallower anterior chamber, and cluster 2 had a deeper anterior chamber and thicker iris. Cluster 3 was characterized by components intermediate of cluster 1 and 2, but had greater iris curvature. AOD500, TISA500, TISA750, AOD750, TISA750 were significantly less in cluster 1 compared with the other clusters (ANOVA p <0.001 for all, p <0.05 for all pairwise comparison). When compared with the other clusters, there was a significant difference between the ACD and ACA values of cluster 1 and the other clusters (ANOVA p <0.001, p <0.05 for all pairwise comparison), with cluster 1 having the smallest mean ACD and ACA. Likewise, LV was greatest in cluster 1 (1111.2±243.1 μm) followed by cluster 3 (992.2±191.8 μm) and 2 (719.8±194.6 μm)(ANOVA p <0.001, p <0.05 for all pairwise comparison). Cluster 2 had the greatest IT2000 (ANOVA p = 0.048, p <0.05 comparison between cluster 1 and cluster2), ITCM (ANOVA p = 0.041, p <0.05 comparison between cluster 1 and cluster2) and iris area (ANOVA p <0.001, p <0.05 for all pairwise comparison). However, iris curvature was found to be greater in cluster 3 (ANOVA p<0.001, p <0.05 for comparison between cluster 3 and the other groups) indicating a higher degree of pupillary block in this group (Fig 2).

**Table 4:** Comparison of angle and anterior segment parameters (Mean ± SD) measured by anterior segment optical coherence tomography and B-scan ultrasonography in each cluster.

	Cluster 1 (43)	cluster 2 (113)	cluster 3 (92)	P-value ^a^	P (1 vs. 2) ^b^	P (1 vs. 3) ^b^	P (2 vs.3) ^b^
Axial length (mm)	21.77±0.77	22.46±0.76	21.82±1.16	<0.001	<0.001	>0.99	<0.001
**Angle Parameters**							
AOD500 (mm)	0.017±0.027	0.084±0.077	0.051±0.054	<0.001	<0.001	0.011	0.001
TISA500 (mm^2^)	0.013±0.022	0.035±0.032	0.031±0.038	<0.001	<0.001	0.003	<0.001
AOD750 (mm)	0.042±0.042	0.155±0.097	0.079±0.068	<0.001	<0.001	0.037	<0.001
TISA750 (mm^2^)	0.023±0.026	0.070±0.050	0.052±0.042	<0.001	<0.001	0.001	<0.001
**Anterior Chamber Parameters**							
ACD (mm)	1.72±0.12	2.21±0.21	1.93±0.19	<0.001	<0.001	<0.001	<0.001
ACW (mm)	11.13±0.50	11.39±0.48	11.21±0.45	0.003	0.029	>0.99	0.010
ACA (mm)	11.87±1.17	15.86±2.13	12.89±1.84	<0.001	<0.001	0.050	<0.001
Lens vault (μm)	1111.2±243.1	719.8±194.6	992.2±191.8	<0.001	<0.001	0.005	<0.001
**Iris Parameters**							
IT750 (mm)	0.45±0.07	0.48±0.08	0.47±0.10	0.294	0.525	0.635	0.859
IT2000 (mm)	0.42±0.08	0.47±0.08	0.44±0.09	0.041	0.045	0.818	0.271
ITCM (mm)	0.58±0.07	0.63±0.19	0.60±0.09	0.048	0.039	0.892	0.121
I-Area (mm^2^)	1.31±0.20	1.62±0.23	1.50±0.23	<0.001	<0.001	<0.001	<0.001
I-Curve(mm)	0.28±0.09	0.27±0.10	0.43±0.08	<0.001	>0.99	<0.001	<0.001
Pupil diameter (mm)	4.22±0.72	4.06±0.82	4.00±0.73	0.297	0.115	0.199	>0.99

APAC: Acute primary angle closure; ACD: Anterior chamber depth; ACW: Anterior chamber width; ACA: Anterior chamber area; AOD: Angle opening distance; CM: Central maximum; IT: Iris thickness; I-Area: Iris area; I-Curve: Iris curvature; PACG: Primary angle closure glaucoma; PACS: Primary angle closure suspect; Primary angle closure suspect; TISA: Trabecular-iris space area

^a^ Statistical significance tested by ANOVA.

^b^Bonferroni post-hoc adjustment for cluster 1 vs. cluster 2; cluster 1 vs cluster 2; cluster 2 vs. cluster 3

**Fig 3:**
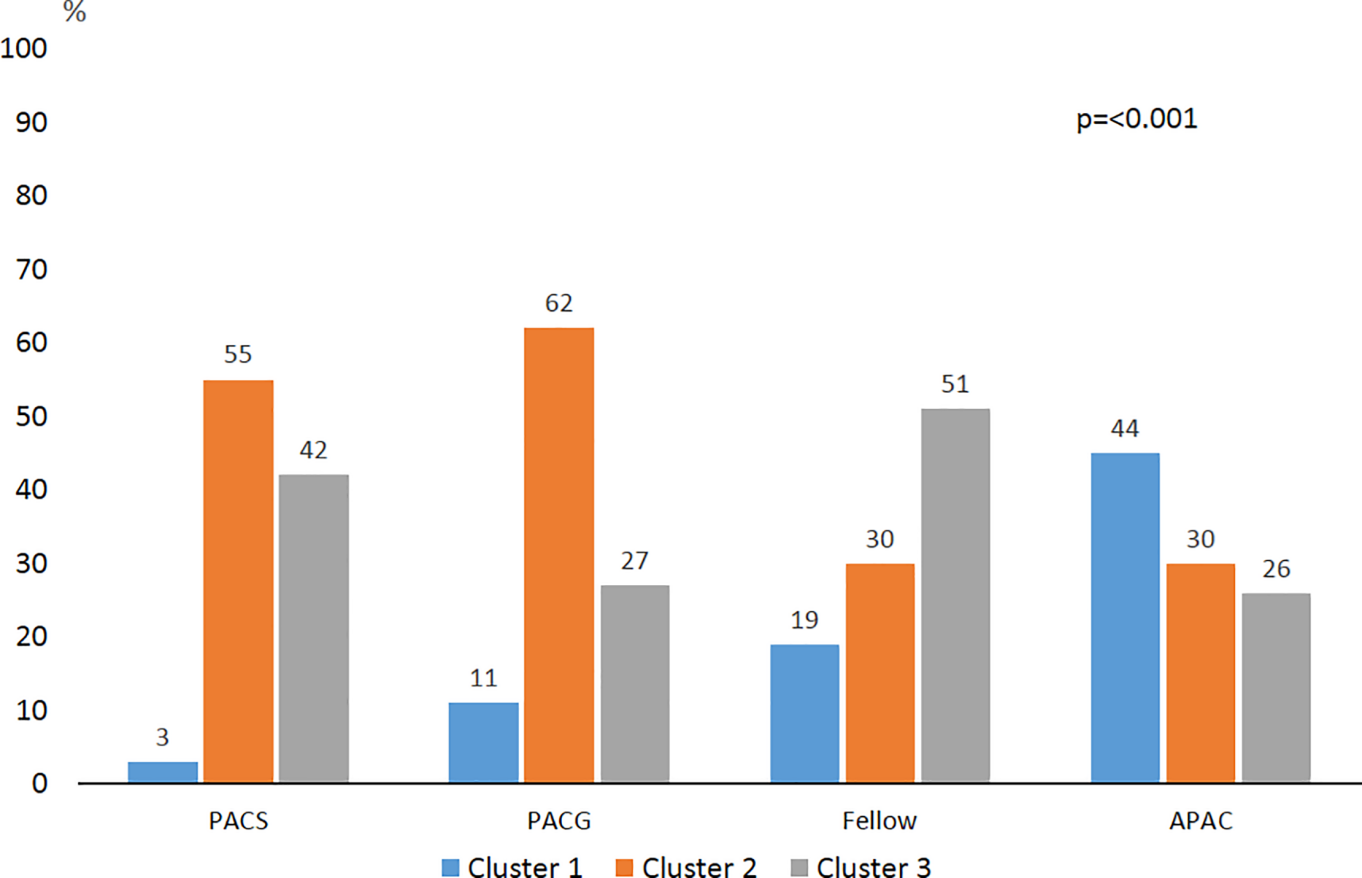
The distribution of clusters in shows a significant difference (*p<0*.*001*) among primary angle closure suspects (PACS) primary angle closure glaucoma (PACG), acute primary angle closure (APAC) eyes and *fellow* eyes of APAC.

There was a statistically significant difference in the distribution of clusters among subtypes of angle closure eyes (p<0.001). Although the pattern of clusters was similar in PACS and PACG eyes with the majority of eyes classified into cluster 2 (55%, and 62%, respectively), cluster 1 (44%) and cluster 3 (51%) had the greatest proportion in the APAC and *fellow* eyes of APAC, respectively. Just 3% of PACS eyes and 11% of PACG eyes were classified in cluster 1 compared to 44% of eyes in APAC and 19% of *fellow* eyes of APAC. Cluster 2 was attributed to most PACG eyes (62%) and PACS eyes (55%) in addition to 30% of APAC eyes and 30% of *fellow* eyes of APAC. Cluster 3 was assigned to 51% of *fellow* eyes in contrast to PACS (42%), PACG (27%), or APAC (26%). (Fig 3)

For internal validation, the same approach was used for half-datasets A and B and similar results were found. (S1 Table). In both datasets, 3 clusters were identified (p = 0.423) with fewer patients classified as cluster 1 and more patients were classified as cluster 2. Similar to our main dataset, cluster 1 was characterized by a shallower anterior chamber, and cluster 2 had a deeper anterior chamber and greater iris area in dataset A and B. Although the difference in IT750 did not reach statistical significance in dataset A, cluster C had a steeper iris in both datasets.

## Discussion

In the present study, three clusters were identified using hierarchical clustering methods. Cluster 1 was characterized by a shallower anterior chamber, and cluster 2 had a deeper anterior chamber and thicker iris. Cluster 3 was characterized by components intermediate of cluster 1 and 2, but had greater iris curvature. There was a significant difference in the distribution of eyes assigned to different clusters among subtypes of angle closure. About half of APAC eyes assigned to cluster 1. While the majority of eyes in the PACG and PACS subtypes contributed to cluster 2, the *fellow* eyes of APAC subjects were assigned most frequently to cluster 3.

With the advent of ASOCT, the entire anterior segment can be captured in a single image to assess angle, iris, and lens parameters. Qualitative and quantitative evaluation of the anterior segment in these eyes helps to elucidate the underlying cause of angle closure.[10–12, 15, 18, 19], consensus is emerging that angle closure is not a single disease entity caused by a single mechanism, but instead a group of disease entities.[7, 20] Categorization of angle closure eyes according to their clinical features is clinically relevant as it may allow the primary treatment modality to be optimized based on the types of angle closure. For example, angle closure due to pupillary block is relieved by LPI.[3] As another example, if forward movement of the iris is the main mechanism of angle closure, it instead should be managed by lens removal.[21, 22] And still as another example, angle closure due to plateau iris configuration is not resolved by LPI and laser peripheral iridoplasty is indicated for these cases.[5, 6]

Recently, hierarchical cluster analysis has been used for classification of angle closure eyes. This method segments a group of patients into clusters such that those within each cluster are more closely related to one another than those attributed to different clusters.[8, 13, 23] The current study produced the same number of clusters as did Nongpiur et al. [13] One cluster (cluster 1) was characterized by a large LV and a small ACD (a predominant LV component). Forward movement of the lens, due to increased lens thickness, may induce an incremental increase of LV and predispose the eye to angle closure, especially in small eye.[8, 13, 23, 24] The second cluster (cluster 2) demonstrated a large iris area and a relatively small LV with deep anterior chambers (a predominant iris component). Previously, a greater iris area has been shown to be independently related to angle closure.[8, 25] The third cluster (cluster3) is comprised of intermediate components of the other two clusters and a high iris curvature (a predominant pupil block). It is well-documented the main mechanisms of angle closure is pupillary block and iris curvature has been proposed to be an indicator of pupillary block.[11, 19, 26]

In the current study, there is a significant difference in the proportion of clusters among subtypes of angle closures. Notably, nearly one half of the APAC was assigned to cluster 1(a predominant LV component), a proportion much greater than in PACS and PACG. This is consistent with earlier investigations on APAC eyes. Moghimi and colleagues evaluated ASOCT images of the eyes with PAC and demonstrated that exaggerated LV was responsible for one half of the APAC eyes.[12]

Although the presence of an increased LV was shown to be strongly associated with angle closure, independently of lens thickness and lens position, a large LV could have a more important role in predisposing eyes to APAC.[18, 19, 27, 28] In cross-sectional studies of Chinese and Iranian populations, investigators showed that APAC eyes have greater LV compared to PACS or PACG. [18, 19, 27, 28] Notably, some portion of the increment of LV in APAC eyes might be due to choroidal expansion before or during the acute attack.[27]

Similar to APAC eyes, the proportion of *fellow* eyes assigned to cluster 1 was greater than in the PACS and PACG eyes. This is in line with previous reports that showed APAC is a bilateral condition and common anatomical characteristics of APAC and fellow eyes predispose the fellow eyes to an acute attack [29] Longitudinal studies are needed to investigate if the PACS or PACG eyes that have features associated with being assigned to cluster 1 are at risk of developing an angle closure attack.

The PACG and PACS eyes were mainly classified into cluster 2 (prominent iris component). This is in line with Beak et al[8] study with the same purpose of sub-classifying PACG and PACS, in which they did not find a significant difference between the two groups regarding their distribution into clusters. Similar to our results, Nongpiur and colleagues found a higher proportion of PACG patients were categorized in the cluster with prominent iris compared with PACS.[23] It is well demonstrated that iris curvature decreases after LPI, while iris area does not;[27, 29, 30] and it can be speculated that larger iris area may be a risk factor for progressive closure of the angle once the pupillary block is eliminated after LPI.[23, 30] The mechanism by which larger iris area contributes to angle closure is possibly explained by the dynamic properties of the iris. [23]

Some limitations of the current study should be considered. Although the study subjects recruited consecutively, PACG group had greater proportion of men compared to other groups. As our findings were not validated in another independent group of subjects, it is not known whether similar results arise when applied to larger populations or other ethnicities. Also, dynamic factors like change in iris volume were not evaluated in this study. Moreover, the current findings only relied on measurements from one meridian, and it is possible that there are meridian-specific differences. Finally, the ciliary body cannot be imaged with ASOCT and, therefore, eyes with plateau iris configuration cannot be further classified.

## Conclusion

It is possible to determine the predominant anatomical component(s) in different subtypes of angle closure. A significant difference in the distribution of clusters among subtypes of angle closure was observed in the present study. Further prospective longitudinal studies are warranted to determine the natural history and the best treatment for each cluster in different subtypes.

## Supporting information

S1 Table Comparison of angle and anterior segment parameters (Mean ± SD) measured by anterior segment optical coherence tomography and B-scan ultrasonography in each cluster identified in dataset A and dataset B. (DOCX)

S2 Table Dataset. (PDF)
