# Supplementary material for: Classification of primary angle closure spectrum with hierarchical cluster analysis
Source: PLoS One. 2018 Jul 23;13(7):e0199157. doi: 10.1371/journal.pone.0199157 (PMC6056027; doi:10.1371/journal.pone.0199157)
Supplement: S1 Table — (DOCX) [file pone.0199157.s001.docx]

**S1 Table - Comparison of angle and anterior segment parameters (Mean ± SD) measured by anterior segment optical coherence tomography and B-scan ultrasonography in each cluster identified in dataset A and dataset B**

|  | **Dataset A** | | | | | **Dataset B** | | | |
| --- | --- | --- | --- | --- | --- | --- | --- | --- | --- |
|  | **Cluster 1 (n=31)** | **cluster 2 (n=52)** | | **cluster 3 (n=39 )** | **P-value*** | **Cluster 1 (n=24)** | **cluster 2 (n=62)** | **cluster 3 (n=40)** | **P-value*** |
| Axial length (mm) | 21.55±0.62 | | 22.42±0.84 | 21.97±1.32 | 0.001 | 21.47±1.49 | 22.27±0.71 | 22.19±0.82 | 0.002 |
| AOD500 (mm) | 0.014±0.027 | | 0.084±0.084 | 0.068±0.058 | <0.001 | 0.017±0.023 | 0.080±0.072 | 0.057±0.054 | <0.001 |
| TISA500 ( mm^2^) | 0.011±0.020 | | 0.037±0.036 | 0.040±0.026 | <0.001 | 0.014±0.017 | 0.033±0.031 | 0.031±0.029 | 0.016 |
| AOD750 (mm) | 0.028±0.038 | | 0.146±0.114 | 0.104±0.075 | <0.001 | 0.040±0.043 | 0.146±0.089 | 0.106±0.068 | <0.001 |
| TISA750 ( mm^2^) | 0.017±0.024 | | 0.070±0.056 | 0.067±0.041 | <0.001 | 0.023±0.022 | 0.068±0.048 | 0.057±0.040 | <0.001 |
| ACD (mm) | 1.74±0.13 | | 2.24±0.27 | 1.96±0.19 | <0.001 | 1.76±0.18 | 2.17±0.19 | 2.00±0.19 | <0.001 |
| ACW (mm) | 11.01±0.37 | | 11.42±0.54 | 11.21±0.51 | 0.001 | 11.15±0.49 | 11.39±0.45 | 11.30±0.44 | 0.084 |
| ACA (mm) | 11.44±1.29 | | 16.01±2.59 | 12.81±1.92 | <0.001 | 11.89±1.28 | 15.49±1.78 | 13.47±1.83 | <0.001 |
| Lens vault (µm) | 1086.0±220.7 | | 734.8±255.2 | 927.9±190.0 | <0.001 | 1129.9±210.8 | 770.7±211.4 | 936.3±206.6 | <0.001 |
| IT750 (mm) | 0.48±0.10 | | 0.49±0.10 | 0.47±0.11 | 0.480 | 0.47±0.07 | 0.48±0.07 | 0.44±0.10 | 0.040 |
| IT2000 (mm) | 0.44±0.17 | | 0.44±0.08 | 0.44±0.06 | 0.694 | 0.43±0.09 | 0.47±0.08 | 0.43±0.07 | 0.075 |
| I-Area (mm^2^) | 1.42±0.32 | | 1.62±0.23 | 1.53±0.25 | 0.009 | 1.45±0.25 | 1.60±0.19 | 1.47±0.23 | 0.001 |
| I-Curve(mm) | 0.27±0.10 | | 0.37±0.12 | 0.43±0.08 | 0.001 | 0.34±0.11 | 0.28±0.10 | 0.40±0.13 | 0.009 |
| Pupil diameter (mm) | 4.42±0.58 | | 4.19±0.90 | 4.02±0.76 | 0.140 | 4.05±0.55 | 4.18±0.62 | 4.00±0.73 | 0.089 |
| APAC: Acute primary angle closure; ACD: Anterior chamber depth; ACW: Anterior chamber width; ACA: Anterior chamber area; AOD: Angle opening distance; CM: Central maximum; IT: Iris thickness; I-Area: Iris area; I-Curve: Iris curvature; PACG: Primary angle closure glaucoma; PACS: Primary angle closure suspect; Primary angle closure suspect; TISA: Trabecular-iris space area  *Statistical significance tested by ANOVA. | | | | | | | | | |
