# Supplementary material for: Classification of primary angle closure spectrum with hierarchical cluster analysis
Source: PLoS One. 2018 Jul 23;13(7):e0199157. doi: 10.1371/journal.pone.0199157 (PMC6056027; doi:10.1371/journal.pone.0199157)
Supplement: S2 Table — (PDF) [file pone.0199157.s002.pdf]

No	Group	Group_NO	ODOS	age	Gender_	ACDUS	preIOP	CTHICK
1.00	attack	4.00	1	52.00	0.00	2.87	20.00	612.20
2.00	attack	4.00	1	68.00	0.00	2.01	12.00	521.40
3.00	attack	4.00	1	48.00	1.00	2.23	11.00	469.00
4.00	attack	4.00	1	54.00	0.00	2.18	5.00	564.60
5.00	attack	4.00	1	53.00	0.00	2.33	12.00	639.50
6.00	attack	4.00	1	72.00	0.00	2.44	20.00	485.70
7.00	attack	4.00	1	58.00	0.00	2.33	12.00	676.80
8.00	attack	4.00	1	80.00	0.00	1.96	9.00	660.60
9.00	attack	4.00	1	46.00	0.00	2.86	13.00	525.20
10.00	attack	4.00	1	79.00	0.00	2.11		591.40
11.00	attack	4.00	1	52.00	1.00	2.28		574.10
12.00	attack	4.00	1	54.00	0.00	2.14	5.00	712.30
13.00	attack	4.00	1	45.00	0.00	2.18	18.00	735.10
14.00	attack	4.00	1	62.00	0.00	2.16	20.00	945.00
15.00	attack	4.00	1	60.00	0.00	2.63	30.00	560.20
16.00	attack	4.00	1	58.00	0.00	2.41	8.00	584.60
17.00	attack	4.00	1	68.00	0.00	2.49	16.00	541.00
18.00	attack	4.00	1	52.00	1.00	2.37	16.00	565.50
19.00	attack	4.00	1	60.00	1.00	2.53	18.00	612.10
20.00	attack	4.00	1	72.00	0.00	2.19	15.00	559.80
21.00	attack	4.00	1	51.00	0.00	2.54	12.00	615.60
22.00	attack	4.00	1	53.00	1.00			609.70
23.00	attack	4.00	1	58.00	0.00	2.46	15.00	564.00
24.00	attack	4.00	1	66.00	1.00	2.60	10.00	534.70
25.00	attack	4.00	1	63.00	0.00	2.11	10.00	514.60
26.00	attack	4.00	1	52.00	1.00	2.76	19.00	533.00
27.00	attack	4.00	1	68.00	0.00	2.13	19.00	538.10
28.00	attack	4.00	1	52.00	0.00	2.07	20.00	589.20
29.00	attack	4.00	1	68.00	1.00	2.69	7.00	584.50
30.00	attack	4.00	1	51.00	0.00		8.00	501.00
31.00	attack	4.00	1	58.00	0.00	2.27	12.00	543.60
32.00	attack	4.00	1	88.00	0.00	2.06	8.00	561.20
33.00	attack	4.00	1	61.00	0.00	2.11	10.00	500.60
34.00	attack	4.00	1	69.00	0.00	2.08	20.00	478.30
35.00	attack	4.00	1	69.00	1.00			615.60
36.00	attack	4.00	1	70.00	0.00	2.09	6.00	518.40
37.00	attack	4.00	1	54.00	0.00	2.39	18.00	596.20
38.00	attack	4.00	1	70.00	1.00	2.54	4.00	552.20
39.00	attack	4.00	1	56.00	0.00	2.29	8.00	559.00
40.00	attack	4.00	1	70.00	0.00	2.26	12.00	479.30
41.00	attack	4.00	1	60.00	1.00	2.37	8.00	549.40
42.00	attack	4.00	1	68.00	1.00			598.60
43.00	attack	4.00	1	75.00	0.00			574.30
44.00	attack	4.00	1	70.00	0.00	2.06	18.00	612.90
45.00	attack	4.00	1	71.00	0.00	2.28	6.00	526.60
46.00	attack	4.00	1	71.00	0.00	2.57	10.00	563.70

47.00	attack	4.00	1	60.00	1.00	1.97	12.00	593.50
48.00	attack	4.00	1	68.00	1.00	2.28	8.00	576.90
49.00	attack	4.00	1	72.00	1.00	2.06	6.00	522.60
50.00	attack	4.00	1	62.00	0.00	2.17	9.00	546.20
51.00	attack	4.00	1	58.00	0.00	2.53	7.00	611.00
52.00	attack	4.00	1	55.00	0.00	2.21	13.00	1309.00
53.00	attack	4.00	1	63.00	0.00	2.56	10.00	596.70
54.00	attack	4.00	1	65.00	0.00	1.98	16.00	450.80
55.00	fellow	3.00	1	58.00	0.00	2.45	8.00	598.10
56.00	fellow	3.00	1	57.00	1.00	2.25	10.00	525.60
57.00	fellow	3.00	1	48.00	1.00	2.44	11.00	496.80
58.00	fellow	3.00	1	68.00	0.00	2.42	16.00	526.40
59.00	fellow	3.00	1	54.00	0.00	2.26	14.00	585.60
60.00	fellow	3.00	1	72.00	0.00	2.54	18.00	509.90
61.00	fellow	3.00	1	65.00	0.00	2.01	16.00	551.20
62.00	fellow	3.00	1	66.00	1.00	2.35	10.00	533.90
63.00	fellow	3.00	1	60.00	0.00	2.65	14.00	567.80
64.00	fellow	3.00	1	66.00	1.00	2.53	16.00	556.80
65.00	fellow	3.00	1	66.00	1.00	2.26	10.00	556.10
66.00	fellow	3.00	1	62.00	0.00	2.39	6.00	679.20
67.00	fellow	3.00	1	60.00	1.00	2.54	11.00	546.20
68.00	fellow	3.00	1	52.00	0.00	2.15	18.00	576.40
69.00	fellow	3.00	1	53.00	0.00	2.29	12.00	631.10
70.00	fellow	3.00	1	51.00	0.00		10.00	511.50
71.00	fellow	3.00	1	63.00	0.00	2.15	12.00	537.70
72.00	fellow	3.00	1	60.00	1.00	2.74	11.00	529.50
73.00	fellow	3.00	1	45.00	0.00	2.20	12.00	585.30
74.00	fellow	3.00	1	60.00	0.00	2.60	14.00	507.60
75.00	fellow	3.00	1	79.00	0.00	2.04		572.60
76.00	fellow	3.00	1	63.00	1.00	2.31	14.00	528.80
77.00	fellow	3.00	1	62.00	0.00	2.48	14.00	523.00
78.00	fellow	3.00	1	72.00	1.00	2.22	12.00	490.20
79.00	fellow	3.00	1	54.00	0.00	1.99	8.00	635.70
80.00	fellow	3.00	1	62.00	0.00	2.49	12.00	590.50
81.00	fellow	3.00	1	51.00	0.00	2.51	12.00	635.20
82.00	fellow	3.00	1	62.00	1.00	2.76	9.00	592.70
83.00	fellow	3.00	1	88.00	0.00	2.15	13.00	561.20
84.00	fellow	3.00	1	45.00	0.00	2.66	12.00	565.40
85.00	fellow	3.00	1	48.00	0.00	2.41	19.00	625.00
86.00	fellow	3.00	1	72.00	0.00	2.23	10.00	533.00
87.00	fellow	3.00	1	53.00	1.00			590.50
88.00	fellow	3.00	1	49.00	1.00	2.47	12.00	478.40
89.00	fellow	3.00	1	70.00	0.00	2.23	6.00	531.40
90.00	fellow	3.00	1	57.00	1.00	2.47	16.00	536.00
91.00	fellow	3.00	1	52.00	1.00	2.90	22.00	584.80
92.00	fellow	3.00	1	58.00	0.00	2.33	12.00	596.80
93.00	fellow	3.00	1	58.00	0.00	2.55	8.00	480.00

94.00	fellow	3.00	1	57.00	0.00		14.00	524.50
95.00	fellow	3.00	1	54.00	0.00		14.00	515.90
96.00	fellow	3.00	1	63.00	0.00	2.96	10.00	558.20
97.00	fellow	3.00	1	54.00	0.00	2.39	18.00	591.80
98.00	fellow	3.00	1	53.00	0.00	2.07	18.00	545.50
99.00	fellow	3.00	1	52.00	1.00	2.32		589.10
100.00	fellow	3.00	1	61.00	0.00	2.04	12.00	506.10
101.00	fellow	3.00	1	55.00	0.00	2.17	13.00	537.00
102.00	fellow	3.00	1	53.00	0.00	2.60	10.00	559.70
103.00	fellow	3.00	1	71.00	0.00	2.45	12.00	523.60
104.00	fellow	3.00	1	70.00	0.00	2.38	10.00	627.30
105.00	fellow	3.00	1	56.00	0.00	2.37	8.00	398.00
106.00	fellow	3.00	1	52.00	0.00	2.82	14.00	625.90
107.00	fellow	3.00	1	46.00	0.00	2.59	13.00	486.90
108.00	PACG	2.00	1	65.00	1.00	2.10	20.00	496.30
109.00	PACG	2.00	1	59.00	0.00	2.94	23.00	571.60
110.00	PACG	2.00	2	54.00	1.00	2.21	20.00	503.50
111.00	PACG	2.00	1	63.00	1.00	2.37	24.00	615.80
112.00	PACG	2.00	2	53.00	0.00	2.75	16.00	632.40
113.00	PACG	2.00	1	59.00	0.00	2.04	10.00	497.10
114.00	PACG	2.00	2	40.00	0.00	2.54	28.00	583.40
115.00	PACG	2.00	1	53.00	1.00	2.25	18.00	505.20
116.00	PACG	2.00	1	42.00	0.00		15.00	530.50
117.00	PACG	2.00	1	60.00	1.00		12.00	481.30
118.00	PACG	2.00	1	55.00	0.00	2.69	19.00	578.80
119.00	PACG	2.00	1	58.00	0.00		19.00	575.80
120.00	PACG	2.00	2	60.00	1.00	2.26	34.00	579.70
121.00	PACG	2.00	2	55.00	1.00		16.00	556.90
122.00	PACG	2.00	1	60.00	1.00	2.82	13.00	616.40
123.00	PACG	2.00	1	52.00	1.00	2.27	26.00	559.90
124.00	PACG	2.00	1	74.00	0.00	2.33	20.00	489.50
125.00	PACG	2.00	1	65.00	0.00	2.68	16.00	562.00
126.00	pACG	2.00	2	59.00	1.00	2.18	24.00	616.00
127.00	PACG	2.00	2	37.00	1.00		18.00	573.20
128.00	PACG	2.00	1	80.00	1.00	2.03	24.00	524.70
129.00	PACG	2.00	2	63.00	0.00		15.00	533.80
130.00	PACG	2.00	1	57.00	0.00	2.39	16.00	532.80
131.00	PACG	2.00	2	73.00	0.00	2.54	14.00	500.40
132.00	PACG	2.00	2	65.00	0.00	2.23	24.00	471.90
133.00	PACG	2.00	2	50.00	0.00	2.47	10.00	523.60
134.00	PACG	2.00	1	56.00	0.00		12.00	559.10
135.00	PACG	2.00	1	52.00	1.00		16.00	603.50
136.00	PACG	2.00	2	54.00	0.00	2.52	18.00	544.70
137.00	PACG	2.00	2	66.00	1.00	1.96	9.00	481.00
138.00	PACG	2.00	1	65.00	0.00		23.00	533.50
139.00	PACG	2.00	2	66.00	1.00		14.00	608.80
140.00	PACG	2.00	2	53.00	0.00		14.00	531.80

141.00	PACG	2.00	1	70.00	1.00		13.00	522.90
142.00	PACG	2.00	1	56.00	1.00	2.66	24.00	550.50
143.00	PACG	2.00	1	59.00	1.00	2.49	18.00	616.50
144.00	PACG	2.00	2	68.00	1.00	2.54	24.00	562.80
145.00	PACG	2.00	2	67.00	1.00	2.57	27.00	539.00
146.00	PACG	2.00	1	74.00	1.00	2.76	20.00	560.50
147.00	PACG	2.00	1	50.00	1.00	2.68	18.00	581.60
148.00	PACG	2.00	1	50.00	0.00	2.68	14.00	506.60
149.00	pACG	2.00	2	54.00	0.00	2.72	50.00	539.50
150.00	PACG	2.00	2	45.00	1.00		19.00	588.00
151.00	PACG	2.00	2	61.00	1.00		19.00	557.70
152.00	PACG	2.00	2	48.00	1.00		12.00	612.20
153.00	PACG	2.00	2	57.00	0.00	2.49	18.00	547.50
154.00	PACG	2.00	1	70.00	0.00	2.67	26.00	632.80
155.00	PACG	2.00	2	86.00	1.00		32.00	525.70
156.00	pACG	2.00	1	53.00	0.00		15.00	532.90
157.00	PACG	2.00	1	64.00	1.00	2.60	17.00	555.80
158.00	PACG	2.00	2	55.00	0.00	2.58	29.00	505.00
159.00	PACG	2.00	2	70.00	1.00		46.00	614.90
160.00	PACG	2.00	2	57.00	0.00	3.00	18.00	568.10
161.00	PACS	1.00	2	58.00	0.00	2.30		546.60
162.00	PACS	1.00	2	78.00	1.00	2.27	18.00	511.60
163.00	PACS	1.00	1	69.00	0.00	3.14	14.00	603.90
164.00	PACS	1.00	1	63.00	1.00	2.60	14.00	539.40
165.00	PACS	1.00	2	54.00	0.00	2.41	16.00	592.00
166.00	PACS	1.00	2	53.00	0.00	2.57	20.00	585.20
167.00	PACS	1.00	1	59.00	0.00	2.46	10.00	526.50
168.00	PACS	1.00	1	60.00	1.00	2.28	21.00	577.40
169.00	PACS	1.00	1	69.00	1.00	2.24	20.00	584.50
170.00	PACS	1.00	2	47.00	0.00	2.64	16.00	537.70
171.00	PACS	1.00	1	65.00	0.00	2.76	14.00	527.60
172.00	PACS	1.00	1	50.00	0.00	2.47	16.00	562.30
173.00	PACS	1.00	1	65.00	1.00		12.00	608.00
174.00	PACS	1.00	2	70.00	0.00	2.64	12.00	554.70
175.00	PACS	1.00	2	71.00	0.00	2.67	20.00	526.40
176.00	PACS	1.00	2	44.00	0.00	2.74	12.00	614.60
177.00	PACS	1.00	2	66.00	1.00	2.52	19.00	541.00
178.00	PACS	1.00	1	64.00	0.00	2.47	14.00	535.00
179.00	PACS	1.00	2	55.00	1.00	2.31	12.00	530.70
180.00	PACS	1.00	1	54.00	0.00		12.00	521.10
181.00	PACS	1.00	1	69.00	0.00	2.25	17.00	545.90
182.00	PACS	1.00	1	54.00	1.00	2.29	12.00	581.50
183.00	PACS	1.00	1	54.00	1.00	2.63	15.00	592.20
184.00	PACS	1.00	1	60.00	0.00	2.59	16.00	638.50
185.00	PACS	1.00	1	81.00	1.00	2.53	13.00	512.30
186.00	PACS	1.00	2	71.00	1.00	2.44	17.00	517.80
187.00	PACS	1.00	1	66.00	0.00	2.46	16.00	533.90

188.00	PACS	1.00	1	62.00	0.00	2.45	14.00	508.70
189.00	PACS	1.00	1	60.00	0.00	2.62	18.00	594.90
190.00	PACS	1.00	1	69.00	0.00		18.00	611.50
191.00	PACS	1.00	1	47.00	1.00	2.60	18.00	504.60
192.00	PACS	1.00	2	60.00	0.00	2.53	18.00	558.00
193.00	PACS	1.00	1	72.00	0.00	2.35	16.00	186.70
194.00	PACS	1.00	1	56.00	0.00	2.93	18.00	480.50
195.00	PACS	1.00	2	53.00	0.00	2.67	17.00	614.60
196.00	PACS	1.00	1	62.00	0.00	2.10	16.00	561.30
197.00	PACS	1.00	1	74.00	0.00	2.16	13.00	537.30
198.00	PACS	1.00	2	49.00	0.00	3.01	16.00	586.70
199.00	PACS	1.00	1	52.00	1.00	2.66		546.30
200.00	PACS	1.00	2	66.00	1.00	2.41	18.00	545.40
201.00	PACS	1.00	2	81.00	1.00	2.55	16.00	
202.00	PACS	1.00	1	46.00	0.00	2.45	20.00	586.40
203.00	PACS	1.00	1	74.00	0.00	2.49	12.00	559.70
204.00	PACS	1.00	2	62.00	0.00	2.79	15.00	505.40
205.00	PACS	1.00	1	69.00	1.00	2.34	16.00	588.00
206.00	PACS	1.00	1	65.00	0.00	2.46	17.00	571.50
207.00	PACS	1.00	2	63.00	1.00		18.00	523.80
208.00	PACS	1.00	1	49.00	1.00	2.29	15.00	580.50
209.00	PACS	1.00	1	49.00	0.00	2.29	15.00	559.70
210.00	PACS	1.00	1	60.00	1.00	2.27	12.00	529.10
211.00	PACS	1.00	2	50.00	0.00	2.33	15.00	495.60
212.00	PACS	1.00	1	57.00	0.00	2.32	13.00	582.10
213.00	PACS	1.00	1	61.00	0.00	2.75	17.00	559.50
214.00	PACS	1.00	1	65.00	0.00	2.61	18.00	668.10
215.00	PACS	1.00	1	70.00	0.00	2.23	20.00	478.30
216.00	PACS	1.00	1	62.00	0.00	2.42		543.50
217.00	PACS	1.00	1	70.00	0.00	2.41	14.00	539.30
218.00	PACS	1.00	2	60.00	0.00	2.49	16.00	524.90
219.00	PACS	1.00	1	56.00	1.00	2.54	12.00	565.90
220.00	PACS	1.00	1	56.00	0.00	2.48	14.00	606.20
221.00	PACS	1.00	2	63.00	0.00	2.27	19.00	552.40
222.00	PACS	1.00	1	56.00	0.00	2.58	12.00	549.30
223.00	PACS	1.00	2	62.00	0.00	2.09	17.00	498.80
224.00	PACS	1.00	1	59.00	0.00	2.35	16.00	505.80
225.00	PACS	1.00	1	72.00	0.00	2.38	18.00	532.20
226.00	PACS	1.00	1	54.00	0.00	1.87	14.00	586.10
227.00	PACS	1.00	1	61.00	1.00	2.55	12.00	531.70
228.00	PACS	1.00	2	67.00	1.00	2.31	16.00	493.20
229.00	PACS	1.00	2	67.00	1.00	3.50	18.00	637.20
230.00	PACS	1.00	1	67.00	0.00	3.10	16.00	563.90
231.00	PACS	1.00	1	66.00	1.00	2.64	18.00	586.60
232.00	PACS	1.00	2	58.00	1.00	3.14	18.00	587.80
233.00	PACS	1.00	1	51.00	1.00	2.41	9.00	513.00
234.00	PACS	1.00	2		0.00		18.00	533.90

235.00	PACS	1.00	1	66.00	1.00	2.47	18.00	483.70
236.00	PACS	1.00	1	65.00	1.00	2.43	10.00	612.20
237.00	PACS	1.00	2	50.00	1.00	2.50	11.00	606.60
238.00	PACS	1.00	2	62.00	1.00		16.00	563.60
239.00	PACS	1.00	2	52.00	0.00	2.50	15.00	506.70
240.00	PACS	1.00	1	45.00	0.00	2.68	12.00	581.20
241.00	PACS	1.00	1	58.00	0.00	2.76	22.00	555.30
242.00	PACS	1.00	1	51.00	1.00	3.02	16.00	555.50
243.00	PACS	1.00	2	62.00	1.00	3.14	21.00	611.90
244.00	PACS	1.00	1	70.00	1.00	2.34	17.00	412.70
245.00	PACS	1.00	1	59.00	1.00	2.64	16.00	508.10
246.00	PACS	1.00	1	39.00	0.00	2.70	14.00	604.70
247.00	PACS	1.00	1	63.00	0.00	2.95		507.50
248.00	PACS	1.00	1	40.00	1.00	2.54	14.00	567.80

CLENGTHmm	AOD250	AOD500	TISA500	ARA	ITCM	TILT	AL	ACDmm
13.94	0.00	0.00	0.00	0.01	0.64	0.15	22.02	2.38
12.38	0.05	0.07	0.04	0.10	0.59	0.23	22.80	1.79
13.44	0.04	0.04	0.03	0.05	0.60	0.19	22.55	1.97
12.71	0.00	0.02	0.00	0.03	0.67	0.26	21.09	1.71
12.24	0.00	0.00	0.00	0.00	0.63	0.24	21.52	1.66
12.67	0.00	0.04	0.01	0.04	0.53	0.22	22.41	2.10
11.73	0.00	0.00	0.00	0.00	0.51	0.20	21.33	1.69
13.43	0.00	0.08	0.01	0.05	0.64	0.30	22.32	1.97
12.97	0.00	0.00	0.00	0.03	0.56	0.20	21.24	2.14
13.18	0.00	0.00	0.01	0.02	0.58	0.32	22.57	1.83
13.55	0.10	0.08	0.07	0.14	0.68	0.32	22.61	1.85
12.46	0.00	0.00	0.00	0.01	0.59	0.32	20.76	1.53
12.38	0.00	0.00	0.01	0.04	0.59	0.26	21.38	1.49
12.42	0.00	0.00	0.00	0.00	0.64	0.21	20.60	1.47
13.07	0.00	0.00	0.01	0.02	0.55	0.24	22.10	2.01
12.38	0.00	0.00	0.00	0.00	0.64	0.20	22.17	1.82
12.37	0.00	0.00	0.00	0.01	0.72	0.25	22.45	1.98
13.67	0.00	0.02	0.00	0.03	0.50	0.21	22.77	2.21
13.41	0.00	0.00	0.00	0.01	0.60	0.28	22.70	1.96
12.08	0.00	0.00	0.00	0.00	0.50	0.27	22.42	1.63
12.92	0.00	0.00	0.00	0.03	0.59	0.21	21.91	2.03
13.19	0.00	0.00	0.00	0.02	0.68	0.22	22.59	2.23
13.55	0.00	0.02	0.00	0.01	0.64	0.23	23.37	2.11
13.59	0.06	0.00	0.03	0.03	0.59	0.19	22.16	2.14
11.80	0.06	0.02	0.03	0.04	0.58	0.30	21.36	1.58
13.09	0.00	0.04	0.01	0.03	0.63	0.25	21.67	2.31
12.69	0.00	0.00	0.00	0.00	0.58	0.29	22.43	1.72
12.27	0.05	0.10	0.04	0.09	0.56	0.26	22.17	1.70
13.60	0.00	0.00	0.00	0.00	0.66	0.18	23.14	2.19
12.87	0.00	0.02	0.00	0.01	0.59	0.26	19.50	1.70
12.16	0.00	0.00	0.00	0.01	0.60	0.15	22.36	1.92
12.75	0.07	0.04	0.04	0.17	0.52	0.25	21.17	1.90
12.13	0.10	0.00	0.04	0.04	0.78	0.34	21.23	1.60
12.89	0.00	0.00	0.00	0.00	0.55	0.30	21.36	1.82
12.42	0.00	0.02	0.01	0.03	0.42	0.20	21.05	1.79
11.95	0.02	0.02	0.02	0.02	0.63	0.27	21.52	1.71
12.65	0.00	0.02	0.02	0.04	0.55	0.26	21.42	2.41
13.89	0.04	0.04	0.02	0.06	0.51	0.33	23.65	1.99
11.78	0.02	0.00	0.02	0.02	0.71	0.24	21.89	1.80
12.88	0.00	0.00	0.00	0.00	0.54	0.26	21.53	1.68
13.31	0.00	0.00	0.00	0.00	0.72	0.24	21.52	1.39
12.79	0.00	0.00	0.00	0.00	1.51		20.66	1.72
14.12	0.00	0.00	0.03	0.38	0.64	0.38	21.36	1.80
13.94	0.00	0.04	0.01	0.04	0.58	0.33	22.58	1.67
13.04	0.00	0.00	0.01	0.01	0.60	0.37	21.85	1.72
14.01	0.08	0.04	0.05	0.06	0.62	0.28	23.00	1.82

13.70	0.00	0.00	0.00	0.00	0.61	0.32	21.89	1.80
12.93	0.00	0.02	0.01	0.02	0.63	0.37	23.01	1.71
13.05	0.00	0.00	0.00	0.01	0.60	0.34	22.67	1.76
13.68	0.03	0.00	0.01	0.02	0.63	0.33	22.82	1.71
12.59	0.02	0.00	0.02	0.02	0.71	0.42	16.12	1.92
12.03	0.00	0.04	0.01	0.04	0.52	0.39	20.63	1.50
13.72	0.00	0.00	0.01	0.01	0.47	0.32	21.93	1.75
13.77	0.00	0.00	0.00	0.01	0.52	0.42	21.33	1.55
11.99	0.07	0.05	0.04	0.05	0.71	0.31	16.08	2.00
12.73	0.00	0.00	0.00	0.01	0.67	0.12	21.23	2.20
13.14	0.02	0.12	0.04	0.10	0.62	0.23	22.83	2.02
12.43	0.00	0.02	0.05	0.06	0.64	0.24	23.30	1.87
12.98	0.00	0.02	0.12	0.13	0.65	0.20	20.66	1.76
12.32	0.04	0.07	0.03	0.06	0.63	0.21	22.51	2.19
13.71	0.00	0.00	0.00	0.03	0.58	0.25	21.18	2.18
13.67	0.00	0.00	0.01	0.02	0.62	0.19	22.64	2.12
13.94	0.00	0.00	0.00	0.00	0.61	0.29	23.41	2.26
13.89	0.07	0.15	0.05	0.11	0.64	0.21	22.69	2.03
13.62	0.00	0.09	0.02	0.06	0.60	0.26	23.65	2.19
12.65	0.00	0.00	0.00	0.00	0.63	0.28	21.09	1.67
13.42	0.00	0.00	0.00	0.01	0.74	0.30	21.74	2.20
12.08	0.00	0.02	0.01	0.03	0.57	0.23	22.29	1.71
12.16	0.00	0.00	0.00	0.00	0.71	0.25	21.80	1.72
13.19	0.00	0.00	0.00	0.00	0.52	0.28	20.22	1.73
12.06	0.02	0.04	0.02	0.03	0.56	0.27	21.05	1.67
14.46	0.00	0.00	0.00	0.01	0.64	0.21	22.38	2.36
12.88	0.04	0.12	0.05	0.24	0.48	0.35	21.45	1.72
13.31	0.00	0.05	0.01	0.02	0.54	0.26	21.70	2.15
13.17	0.00	0.02	0.01	0.02	0.52	0.23	22.26	2.05
12.46	0.00	0.04	0.00	0.02	0.64	0.29	21.91	1.90
12.37	0.02	0.00	0.02	0.05	0.58	0.25	21.79	1.88
12.86	0.00	0.00	0.00	0.01	0.56	0.34	22.45	1.69
13.54	0.06	0.00	0.05	0.07	0.58	0.24	21.37	1.92
13.82	0.11	0.09	0.06	0.12	0.60	0.28	22.86	2.04
12.90	0.00	0.04	0.01	0.03	0.58	0.24	21.86	2.01
13.69	0.00	0.00	0.00	0.02	0.58	0.22	23.06	2.20
12.75	0.07	0.04	0.04	0.17	0.52	0.25	21.31	1.90
13.42	0.05	0.07	0.03	0.06	0.63	0.28	21.39	2.23
12.95	0.00	0.00	0.00	0.00	0.80	0.26	21.67	1.94
13.13	0.00	0.00	0.00	0.00	0.68	0.38	22.33	1.78
13.40	0.06	0.04	0.03	0.09	0.59	0.23	22.54	2.24
13.49	0.00	0.00	0.01	0.02	0.63	0.27	21.96	1.99
12.25	0.02	0.00	0.01	0.02	0.67	0.31	21.71	1.75
13.11	0.00	0.00	0.00	0.00	0.55	0.29	21.52	2.00
13.33	0.02	0.00	0.02	0.03	0.60	0.36	21.54	2.25
11.83	0.03	0.06	0.04	0.06	0.55	0.26	21.46	1.84
13.52	0.05	0.06	0.04	0.06	0.55	0.31	21.59	1.94

12.17	0.00	0.02	0.00	0.01	0.55	0.29	21.05	1.78
12.64	0.00	0.00	0.00	0.00	0.76	0.40	20.87	1.86
14.08	0.02	0.02	0.03	0.24	0.56	0.26	21.64	2.37
12.75	0.00	0.02	0.02	0.04	0.52	0.27	21.42	1.94
11.94	0.00	0.00	0.00	0.01	0.57	0.20	20.84	1.70
13.35	0.07	0.11	0.06	0.30	0.64	0.12	22.63	1.94
12.93	0.04	0.04	0.08	0.09	0.82	0.38	21.34	1.77
12.71	0.00	0.06	0.01	0.05	0.48	0.34	20.64	1.69
13.98	0.04	0.02	0.02	0.03	0.68	0.30	23.01	1.84
13.49	0.02	0.00	0.07	0.08	0.73	0.46	21.93	1.78
13.76	0.00	0.00	0.00	0.00	0.58	0.25	22.15	1.88
11.35	0.06	0.00	0.03	0.05	0.67	0.21	21.96	1.74
13.86	0.00	0.02	0.00	0.01	0.69	0.16	21.54	2.40
12.72	0.00	0.06	0.01	0.05	0.60	0.22	21.43	2.14
13.68	0.00	0.02	0.00	0.03	0.58	0.20	22.92	2.24
13.80	0.10	0.25	0.07	0.16	0.62	0.21	22.06	2.61
12.30	0.00	0.00	0.00	0.01	0.59	0.20	21.93	1.83
12.42	0.04	0.13	0.05	0.18	0.52	0.26	22.14	1.92
13.15	0.10	0.11	0.07	0.12	0.56	0.28	22.03	2.16
12.61	0.00	0.00	0.01	0.04	0.50	0.13	20.87	1.86
13.39	0.00	0.06	0.01	0.04	0.60	0.18	22.54	2.10
12.31	0.00	0.04	0.01	0.03	0.47	0.21	21.22	1.96
13.30	0.03	0.11	0.03	0.07	0.66	0.20	22.04	2.18
12.71	0.00	0.00	0.00	0.00	0.52	0.21	21.05	1.97
13.67	0.04	0.13	0.04	0.11	0.62	0.28	22.95	2.22
12.92	0.02	0.04	0.01	0.04	0.55	0.24	21.52	2.05
13.08	0.04	0.06	0.03	0.07	0.48	0.21	23.43	1.85
13.36	0.00	0.00	0.01	0.02	0.61	0.24	22.45	1.78
14.25	0.00	0.03	0.01	0.02	0.61	0.18	24.83	2.34
13.74	0.00	0.00	0.00	0.02	0.50	0.24	22.69	2.13
12.25	0.00	0.00	0.00	0.01	0.58	0.26	22.76	1.92
12.85	0.06	0.10	0.05	0.09	0.51	0.24	21.62	1.90
12.12	0.00	0.00	0.00	0.00	0.61	0.33	22.93	1.54
13.75	0.00	0.00	0.00	0.01	0.55	0.24	23.37	2.11
12.04	0.02	0.02	0.01	0.02	0.59	0.30	21.93	1.65
14.39	0.02	0.00	0.01	0.02	0.75	0.23	23.35	2.41
12.31	0.00	0.00	0.00	0.01	0.60	0.25	21.68	1.93
13.37	0.00	0.11	0.02	0.06	0.50	0.24	21.93	2.15
12.28	0.00	0.00	0.01	0.01	0.64	0.25	21.94	1.83
13.27	0.00	0.05	0.01	0.03	0.50	0.28	22.02	2.01
12.93	0.04	0.09	0.03	0.06	0.56	0.19	23.22	1.99
13.11	0.11	0.07	0.05	0.08	0.52	0.21	22.45	2.26
13.22	0.02	0.04	0.02	0.05	0.47	0.28	21.85	1.86
12.67	0.05	0.07	0.03	0.06	0.47	0.30	22.18	1.50
13.95	0.00	0.00	0.00	0.02	0.59	0.36	22.50	1.70
13.60	0.02	0.00	0.01	0.02	0.63	0.29	21.43	1.92
13.76	0.04	0.02	0.02	0.02	0.70	0.30	22.64	2.05

13.73	0.13	0.13	0.09	0.22	0.61	0.40	23.37	2.07
13.08	0.00	0.06	0.00	0.05	0.50	0.12	23.58	2.35
13.32	0.04	0.12	0.03	0.09	0.52	0.16	23.31	2.24
12.84	0.00	0.00	0.00	0.02	0.67	0.15	22.06	2.19
13.01	0.00	0.10	0.02	0.08	0.71	0.22	23.15	2.24
13.34	0.02	0.11	0.03	0.08	0.60	0.17	22.52	2.36
13.55	0.02	0.11	0.02	0.07	0.63	0.13	23.74	2.38
12.73	0.05	0.14	0.04	0.11	0.62	0.06	22.22	2.38
12.88	0.00	0.00	0.00	0.01	0.55	0.15	22.40	2.30
14.09	0.06	0.17	0.04	0.09	0.59	0.12	24.10	2.37
13.61	0.06	0.16	0.04	0.11	0.54	0.11	22.19	2.44
12.18	0.02	0.11	0.02	0.06	0.56	0.14	22.27	2.02
13.01	0.00	0.00	0.00	0.01	0.60	0.11	24.21	2.41
13.29	0.00	0.06	0.02	0.05	0.76	0.13	23.12	2.21
14.60	0.09	0.16	0.07	0.42	0.68	0.16	24.10	2.92
13.18	0.02	0.12	0.04	0.10	0.52	0.15	23.56	2.30
13.35	0.05	0.09	0.04	0.07	0.61	0.20	22.27	2.38
12.15	0.00	0.04	0.01	0.03	0.52	0.16	22.49	2.03
13.47	0.20	0.14	0.12	0.29	0.76	0.24	23.15	2.41
13.50	0.04	0.07	0.03	0.08	0.67	0.17	22.42	2.59
12.85	0.02	0.06	0.02	0.05	0.52	0.26	22.34	1.90
12.60	0.06	0.06	0.04	0.25	0.70	0.23	21.89	1.90
14.08	0.15	0.37	0.11	0.23	0.55	0.24	22.00	2.65
13.38	0.07	0.12	0.05	0.25	0.53	0.29	23.31	2.26
12.42	0.12	0.16	0.07	0.17	0.56	0.24	21.94	1.98
13.00	0.00	0.00	0.00	0.00	0.61	0.30	21.63	1.81
13.26	0.04	0.04	0.04	0.09	0.64	0.19	22.39	2.10
13.14	0.04	0.04	0.03	0.06	0.48	0.22	23.19	1.85
13.58	0.06	0.10	0.06	0.32	0.67	0.40	23.01	2.09
13.53	0.05	0.17	0.06	0.12	0.54	0.28	22.90	2.10
13.05	0.08	0.15	0.07	0.14	0.66	0.22	21.56	2.48
13.21	0.04	0.08	0.03	0.07	0.54	0.18	22.54	2.12
13.77	0.00	0.06	0.02	0.06	0.66	0.22	23.35	2.35
13.05	0.02	0.04	0.01	0.03	0.55	0.19	21.88	2.12
13.40	0.00	0.06	0.03	0.07	0.60	0.23	22.21	2.16
13.93	0.11	0.14	0.05	0.10	0.59	0.21	21.45	2.18
14.05	0.06	0.11	0.04	0.11	0.46	0.23	22.45	2.21
12.59	0.09	0.14	0.07	0.11	0.54	0.20	21.86	2.09
11.60	0.04	0.08	0.04	0.09	0.46	0.23	21.12	1.80
12.23	0.24	0.27	0.11	0.21	0.48	0.26	21.39	2.21
12.63	0.11	0.08	0.06	0.20	0.59	0.34	21.79	1.92
12.31	0.02	0.06	0.03	0.08	0.44	0.20	22.45	1.80
13.63	0.04	0.09	0.03	0.06	0.63	0.31	20.67	2.04
13.19	0.10	0.02	0.05	0.08	0.63	0.28	22.62	2.13
12.80	0.11	0.17	0.07	0.18	0.55	0.22	22.41	2.16
12.47	0.06	0.10	0.05	0.10	0.48	0.21	23.07	1.96
13.79	0.06	0.14	0.05	0.10	0.56	0.27	22.86	1.98

13.51	0.00	0.06	0.01	0.05	0.59	0.28	22.26	1.98
13.15	0.00	0.04	0.01	0.02	0.56	0.23	22.20	2.03
13.28	0.00	0.00	0.00	0.00	0.72	0.22	21.52	2.03
13.01	0.10	0.13	0.07	0.11	0.56	0.25	22.21	2.24
13.06	0.00	0.07	0.01	0.03	0.60	0.26	22.32	2.06
12.75	0.16	0.20	0.11	0.52	0.68	0.45	22.59	2.03
13.42	0.00	0.04	0.01	0.04	0.66	0.27	22.80	2.26
13.49	0.11	0.13	0.06	0.13	0.55	0.24	22.60	2.19
12.01	0.08	0.06	0.04	0.10	0.48	0.25	21.31	1.72
13.22	0.15	0.13	0.09	0.30	0.61	0.31	22.09	1.90
14.78	0.24	0.24	0.11	0.20	0.48	0.22	22.60	2.57
13.52	0.05	0.08	0.05	0.08	0.64	0.24	22.50	2.11
13.14	0.00	0.05	0.01	0.03	0.56	0.19	23.01	1.97
		0.14	0.07				21.93	2.12
13.77	0.04	0.08	0.04	0.14	0.68	0.21	21.96	2.15
12.76	0.04	0.04	0.02	0.05	0.64	0.29	21.65	2.08
14.33	0.18	0.15	0.12	0.54	0.71	0.49	21.40	2.40
14.29	0.00	0.00	0.01	0.03	0.60	0.28	23.48	1.99
13.73	0.13	0.19	0.10	0.36	0.62	0.37	21.54	2.16
12.87	0.09	0.07	0.05	0.12	0.48	0.31	21.71	1.73
12.91	0.08	0.02	0.03	0.04	0.70	0.31	21.42	2.04
13.48	0.10	0.10	0.06	0.09	0.62	0.32	20.48	1.97
12.74	0.12	0.12	0.07	0.12	0.63	0.24	21.72	1.80
12.95	0.16	0.19	0.10	0.16	0.50	0.28	20.04	1.93
12.42	0.04	0.06	0.04	0.08	0.65	0.31	21.42	2.04
13.02	0.05	0.07	0.05	0.07	0.80	0.28	22.29	2.34
13.29	0.00	0.00	0.01	0.03	0.56	0.26	21.24	2.11
12.79	0.11	0.11	0.08	0.14	0.68	0.34	21.43	2.04
12.63	0.17	0.18	0.10	0.16	0.71	0.38	21.82	1.96
14.11	0.02	0.00	0.01	0.02	0.63	0.29	22.30	2.14
13.39	0.11	0.15	0.07	0.13	0.55	0.30	21.89	2.07
13.17	0.00	0.09	0.01	0.05	0.55	0.21	22.78	1.89
12.71	0.03	0.03	0.03	0.05	0.67	0.29	20.22	1.91
13.70	0.25	0.20	0.12	0.21	0.60	0.35	21.63	1.71
14.33	0.02	0.08	0.03	0.05	0.56	0.34	21.68	2.25
12.54	0.05	0.05	0.04	0.06	0.50	0.43	19.50	1.77
13.61	0.02	0.02	0.03	0.05	0.60	0.39	21.82	2.01
12.96	0.00	0.02	0.01	0.02	0.63	0.33	21.64	2.02
12.35	0.02	0.02	0.02	0.03	0.77	0.43	21.16	1.53
12.46	0.00	0.05	0.01	0.05	0.70	0.16	23.06	1.92
11.14	0.02	0.13	0.03	0.09	0.59	0.16	21.26	1.94
13.39	0.05	0.21	0.04	0.12	0.63	0.09	22.76	2.85
14.13	0.00	0.00	0.00	0.00	0.76	0.17	23.80	2.62
12.68	0.19	0.26	0.11	0.22	0.68	0.19	22.93	2.03
13.38	0.04	0.09	0.03	0.08	0.72	0.14	24.35	2.59
12.64	0.17	0.22	0.09	0.18	0.74	0.16	21.74	2.23
13.26	0.04	0.09	0.05	0.17	0.44	0.21	22.52	2.36

12.32	0.08	0.16	0.06	0.13	0.79	0.21	21.74	2.35
12.94	0.07	0.07	0.03	0.06	0.57	0.11	21.41	2.18
12.72	0.07	0.11	0.04	0.25	0.54	0.20	23.52	2.09
13.75	0.14	0.08	0.06	0.12	0.54	0.20	22.52	2.41
13.65	0.20	0.25	0.10	0.48	0.43	0.20	21.72	2.54
13.65	0.00	0.04	0.02	0.05	0.68	0.16	23.43	2.20
13.29	0.19	0.21	0.09	0.20	0.58	0.19	21.69	2.56
13.47	0.17	0.22	0.09	0.20	0.58	0.22	22.96	2.56
13.35	0.09	0.14	0.06	0.13	0.73	0.16	23.36	2.63
12.99	0.00	0.00	0.00	0.01	0.60	0.19	21.79	2.32
13.31	0.00	0.05	0.01	0.04	0.62	0.19	22.89	2.34
13.40	0.12	0.14	0.08	0.14	0.65	0.22	22.10	2.47
13.18	0.09	0.19	0.07	0.14	0.57	0.14	22.50	2.65
12.60	0.14	0.21	0.08	0.16	0.67	0.18	21.63	2.06

ACWmm	ACA	ACV	PCCURVmr	ACCURVmr	LENSVAULT	PUPILDmm	AOD750	TISA750
12.01	18.29	118.64	6.64	7.44	684.30	2.69	0.08	0.01
10.84	11.86	68.15	6.08	7.36	772.00	3.23	0.15	0.07
11.67	13.81	84.68	6.75	7.65	902.60	3.23	0.10	0.05
11.10	12.22	72.36	6.75	7.71	869.80	4.99	0.20	0.03
10.85	11.06	61.85	6.61	7.39	874.60	5.07	0.00	0.00
10.64	14.43	83.78	5.63	6.56	756.60	3.84	0.11	0.04
10.32	10.79	58.24	6.13	7.28	713.80	4.35	0.05	0.00
11.63	13.42	80.78	6.45	7.33	927.00	4.11	0.10	0.05
11.07	15.06	89.40	6.51	7.09	756.50	4.53	0.13	0.03
11.35	12.64	74.74	6.21	7.28	1073.00	4.37	0.04	0.02
11.76	14.48	95.62	6.85	7.68	1179.00	4.75	0.15	0.10
10.92	10.68	62.41	6.85	7.36	1097.00	4.91	0.06	0.01
11.00	10.58	63.01	6.43	7.44	1086.00	4.56	0.04	0.02
11.19	9.69	53.42	6.69	6.99	950.50	4.40	0.00	0.00
11.22	13.96	83.25	6.08	7.12	844.00	4.32	0.05	0.02
10.83	12.20	68.27	6.21	8.08	912.00	4.43	0.00	0.00
10.71	12.63	68.82	6.43	7.57	795.70	4.11	0.02	0.01
11.72	16.63	107.04	6.96	8.03	883.70	4.43	0.15	0.03
11.69	14.27	88.89	6.61	8.00	1053.00	5.17	0.06	0.01
10.73	10.40	55.56	6.51	7.20	926.20	3.89	0.00	0.00
11.18	13.83	80.45	6.48	7.52	750.40	4.29	0.10	0.02
11.28	15.61	92.69	6.21	7.09	734.60	4.53	0.08	0.02
11.81	14.75	87.63	6.88	7.47	755.60	4.51	0.02	0.01
11.91	14.54	86.82	6.80	7.65	788.40	3.44	0.00	0.03
10.36	9.69	49.36	6.08	7.25	946.90	4.19	0.02	0.04
11.04	16.11	96.57	5.76	6.72	835.40	4.56	0.11	0.03
11.09	11.58	65.95	6.61	7.44	1023.00	4.69	0.00	0.00
10.76	10.70	59.49	6.19	7.15	854.00	3.39	0.08	0.06
11.82	15.16	91.02	6.35	7.76	846.90	3.52	0.02	0.00
11.25	11.99	70.71	6.91	7.92	1045.00	4.43	0.02	0.01
10.67	11.37	57.76	6.05	7.15	685.30	5.11	0.06	0.01
11.05	12.60	76.51	6.19	7.01	920.90	3.15	0.11	0.07
10.74	9.32	46.63	6.43	7.52	925.10	3.33	0.00	0.04
11.18	12.53	72.09	6.27	7.15	1070.00	4.99	0.04	0.00
10.85	10.51	57.74	4.40	4.99	759.00	5.11	0.08	0.03
10.62	9.90	51.20	6.37	7.36	738.20	4.73	0.00	0.02
10.87	12.23	64.06	4.61	5.44	917.30	4.41	0.09	0.04
11.84	13.43	82.45	6.67	7.28	1156.00	3.09	0.13	0.06
10.43	10.89	56.10	5.84	6.99	740.80	3.36	0.00	0.02
11.24	11.09	63.10	6.56	7.17	1186.00	3.79	0.02	0.00
11.13	7.73	35.00	6.19	7.12	1118.00	3.57	0.00	0.00
11.38	10.37	56.50	7.15	8.19	1259.00	4.90	0.00	0.00
11.96	13.37	83.95	6.59	7.57	1388.00	5.52	0.02	0.03
11.87	12.55	83.55	6.40	7.12	1504.00	3.79	0.11	0.04
11.28	12.08	71.66	6.61	7.15	1276.00	4.64	0.00	0.01
12.16	13.82	86.58	6.56	7.47	1294.00	5.33	0.04	0.06

11.85	13.02	79.39	6.24	7.31	1253.00	4.19	0.00	0.00
11.18	11.05	62.38	6.11	7.09	1222.00	3.47	0.02	0.02
11.27	11.53	67.09	6.32	7.95	1286.00	3.92	0.00	0.00
11.77	11.72	69.22	6.45	7.25	1395.00	3.76	0.02	0.02
10.50	12.19	65.46	5.49	6.45	1246.00	3.87	0.00	0.02
11.02	10.99	65.33	8.13	6.96	1612.00	3.33	0.15	0.04
12.04	12.74	80.98	8.03	6.72	1650.00	7.60	0.02	0.01
11.45	11.50	71.88	5.65	8.35	1691.00	4.40	0.05	0.01
11.38	11.29	57.77	5.33	7.65	1040.00	2.27	0.07	0.05
10.78	16.18	96.54	5.87	6.96	761.60	6.00	0.08	0.01
11.51	14.00	86.26	6.59	7.65	782.70	2.99	0.20	0.09
10.83	12.40	70.55	6.05	7.33	791.20	3.49	0.08	0.06
11.33	11.84	67.17	6.64	7.33	1081.00	4.72	0.02	0.13
10.54	14.60	83.31	6.00	6.85	700.80	4.27	0.10	0.06
11.53	16.36	100.59	5.68	6.43	1139.00	3.89	0.16	0.03
11.85	14.81	91.92	6.40	7.28	792.10	3.89	0.02	0.02
11.98	16.22	102.88	6.80	7.28	826.80	5.31	0.08	0.00
11.93	15.45	98.41	6.77	7.47	1041.00	4.72	0.26	0.11
11.58	16.81	108.52	6.21	7.20	918.10	5.17	0.16	0.06
11.04	11.50	65.58	5.97	6.51	1144.00	4.75	0.00	0.00
11.40	15.70	96.92	6.11	6.88	1007.00	4.08	0.07	0.01
10.57	11.08	59.75	6.64	7.47	773.70	4.45	0.08	0.03
10.78	10.78	57.38	6.77	7.60	767.60	4.43	0.00	0.00
11.52	11.85	69.57	6.35	7.39	1056.00	4.13	0.00	0.00
10.47	10.63	57.54	5.92	6.99	935.90	4.29	0.04	0.03
12.37	16.84	104.72	6.11	7.23	1041.00	4.05	0.02	0.01
11.10	11.79	73.68	6.40	6.85	1107.00	4.40	0.10	0.07
11.36	15.01	89.95	6.08	7.01	988.50	4.11	0.04	0.02
11.27	13.77	81.62	6.37	7.28	976.70	3.55	0.04	0.02
10.74	11.94	65.37	6.13	7.33	957.50	3.68	0.08	0.02
10.62	11.23	58.40	5.97	7.04	918.20	3.23	0.06	0.04
11.07	10.77	60.57	6.64	7.55	1186.00	3.57	0.07	0.01
11.73	13.33	79.90	6.53	7.49	1043.00	4.11	0.00	0.05
11.81	15.44	100.04	6.77	7.39	1065.00	4.59	0.15	0.11
11.16	13.92	81.61	6.24	7.15	797.20	4.69	0.08	0.03
11.76	14.75	88.64	6.37	7.36	877.50	2.88	0.06	0.02
11.05	12.60	76.51	6.19	7.01	920.90	3.23	0.11	0.07
11.15	16.34	100.96	6.27	7.07	933.80	4.99	0.11	0.06
11.23	13.12	75.01	6.64	7.31	1066.00	4.53	0.00	0.00
11.39	12.19	69.76	6.35	7.25	1170.00	4.93	0.00	0.00
11.55	15.77	96.40	6.40	7.39	700.90	4.77	0.06	0.05
11.69	13.10	75.37	5.84	6.85	1003.00	3.87	0.04	0.02
10.69	10.33	53.06	6.21	7.15	814.00	3.44	0.04	0.02
11.17	12.43	69.40	5.79	6.69	1033.00	3.09	0.02	0.00
11.15	15.47	91.13	6.19	6.75	897.30	4.40	0.00	0.02
10.30	10.49	52.92	6.03	7.15	769.50	2.75	0.02	0.06
11.58	13.14	79.23	6.32	7.09	1100.00	3.36	0.05	0.06

10.38	11.12	60.11	5.89	6.72	1139.00	3.55	0.00	0.00
10.91	11.91	62.83	6.19	7.36	1138.00	4.64	0.02	0.00
11.87	17.40	113.93	6.75	7.33	954.00	3.17	0.05	0.05
10.86	11.79	67.23	6.27	7.28	1099.00	1.71	0.08	0.04
10.57	9.55	47.22	5.57	6.51	808.00	2.75	0.02	0.01
11.62	15.06	101.56	6.88	7.79	1016.00	4.35	0.11	0.10
11.31	10.71	55.12	6.48	7.49	1140.00	4.00	0.02	0.09
10.84	11.89	72.44	6.08	7.23	1319.00	3.44	0.17	0.05
12.08	14.77	98.22	6.83	7.47	1224.00	5.55	0.08	0.03
11.71	11.70	66.69	6.08	6.99	1310.00	4.48	0.00	0.08
11.87	12.19	71.32	6.53	7.65	1437.00	4.61	0.02	0.00
10.14	11.10	60.00	6.43	9.33	569.00	3.97	0.04	0.03
11.97	17.47	108.75	7.01	8.05	605.30	4.32	0.04	0.01
10.96	13.75	77.09	6.11	7.09	664.40	3.41	0.13	0.05
11.67	15.96	102.04	6.53	7.55	749.20	2.93	0.15	0.03
11.42	19.61	128.81	6.00	6.88	817.60	3.65	0.34	0.16
10.77	12.68	73.33	6.11	7.25	879.20	4.59	0.06	0.01
10.67	13.65	85.87	6.13	7.23	896.70	3.55	0.17	0.09
11.20	16.58	106.89	6.51	7.12	905.20	4.69	0.21	0.12
10.94	13.34	80.39	6.19	7.52	856.40	4.56	0.18	0.04
11.46	15.69	99.86	6.35	7.28	786.70	4.67	0.13	0.04
10.56	12.68	71.28	6.08	7.20	723.80	3.79	0.13	0.03
11.41	15.70	97.41	6.75	7.71	781.80	4.48	0.18	0.07
11.13	13.51	80.55	6.21	7.15	869.90	3.68	0.04	0.00
11.63	16.86	111.54	7.07	7.47	915.90	4.03	0.16	0.08
11.20	15.10	92.57	6.40	7.20	822.40	4.88	0.12	0.04
11.46	12.39	74.58	6.75	7.76	868.00	2.75	0.07	0.05
11.80	13.14	80.87	6.83	7.63	990.50	4.93	0.05	0.02
12.23	16.36	102.18	6.83	7.92	880.50	3.09	0.07	0.02
11.81	16.22	105.27	6.93	7.31	959.70	4.61	0.08	0.02
10.57	12.84	71.70	6.40	7.12	816.70	4.40	0.04	0.01
11.08	12.17	72.66	5.89	6.77	1010.00	2.56	0.19	0.09
10.64	9.81	52.94	6.08	6.83	935.70	4.16	0.00	0.00
11.82	14.66	87.40	6.35	7.52	948.40	4.77	0.06	0.01
10.49	9.90	52.81	6.03	6.91	991.80	4.60	0.04	0.02
12.27	18.12	114.75	6.24	7.28	957.30	5.97	0.09	0.02
10.68	11.99	65.56	6.13	7.09	824.60	3.17	0.07	0.01
11.43	14.39	85.17	6.43	7.23	881.10	3.31	0.15	0.06
10.70	11.40	61.66	5.81	6.85	939.30	3.44	0.03	0.01
11.26	14.12	85.59	6.27	7.04	1064.00	3.63	0.11	0.03
11.33	12.49	72.53	6.83	7.65	699.90	2.99	0.13	0.06
11.17	13.34	81.71	5.81	6.59	736.20	1.09	0.12	0.08
11.27	10.93	64.82	6.35	7.44	1122.00	4.61	0.10	0.05
11.02	10.99	65.33	6.27	7.12	1294.00	3.68	0.10	0.06
11.90	12.08	71.48	6.11	6.91	1447.00	5.04	0.02	0.02
11.63	12.12	72.58	5.87	6.77	1265.00	3.26	0.06	0.02
11.65	13.28	78.62	6.05	6.96	1227.00	3.39	0.02	0.02

11.44	14.55	95.19	6.19	6.67	1358.00	3.17	0.20	0.14
11.18	16.57	103.26	6.24	7.09	571.90	2.83	0.24	0.05
11.60	16.13	102.87	6.83	7.52	559.90	3.49	0.23	0.09
11.08	15.05	87.20	5.92	7.57	582.60	4.45	0.06	0.02
11.15	15.47	95.17	6.03	6.83	582.10	3.31	0.23	0.08
11.55	16.86	105.48	6.88	7.60	582.10	3.84	0.21	0.08
11.66	17.62	113.43	6.67	7.73	606.40	3.95	0.14	0.07
10.99	17.22	106.68	6.37	7.31	429.20	4.67	0.33	0.11
11.08	15.88	93.69	6.21	7.01	613.80	3.57	0.09	0.01
12.51	18.80	127.95	7.60	7.71	500.30	5.07	0.16	0.09
11.69	18.58	120.70	6.11	6.91	648.40	4.77	0.29	0.10
10.56	13.31	76.09	7.09	7.17	452.30	3.36	0.20	0.06
11.39	15.91	92.28	6.21	7.57	348.60	3.36	0.06	0.01
11.53	15.31	90.57	7.12	7.79	628.20	4.29	0.14	0.05
12.43	23.64	170.76	6.91	7.60	410.00	4.77	0.21	0.11
11.47	16.20	100.38	6.40	7.23	558.70	3.81	0.24	0.10
11.36	16.54	101.35	6.53	7.07	623.80	3.49	0.15	0.07
10.67	12.70	70.95	6.45	7.33	480.10	2.67	0.16	0.03
11.68	17.23	109.13	6.45	7.33	528.70	4.72	0.12	0.14
11.38	17.83	109.93	6.27	7.01	585.70	3.49	0.27	0.08
11.08	13.19	79.29	6.05	7.33	895.10	3.60	0.14	0.05
10.99	12.60	77.54	6.45	7.49	772.50	3.71	0.18	0.08
11.66	20.60	143.49	6.05	6.93	863.90	1.60	0.51	0.23
11.53	16.09	108.42	6.99	7.73	819.90	4.35	0.20	0.09
10.83	12.95	76.95	6.16	7.17	685.70	3.31	0.20	0.13
11.31	13.48	81.95	6.51	7.23	1066.00	5.73	0.00	0.00
11.52	15.38	96.49	6.80	7.47	740.40	4.37	0.10	0.07
11.53	12.38	74.46	6.72	7.79	919.90	2.75	0.04	0.05
11.82	15.11	99.99	7.01	7.84	934.20	5.23	0.14	0.09
11.76	16.05	104.93	6.51	7.15	847.10	4.83	0.21	0.11
11.05	16.64	101.32	6.03	6.75	677.30	2.85	0.14	0.13
11.47	15.35	94.73	6.51	7.49	742.70	4.93	0.12	0.06
11.79	16.75	104.34	6.05	6.51	737.60	4.35	0.11	0.04
11.23	14.40	84.11	6.40	7.17	772.40	4.37	0.06	0.03
11.56	16.14	100.96	6.37	7.15	769.40	5.39	0.13	0.07
12.03	15.91	103.49	6.72	7.17	917.70	3.65	0.19	0.10
11.97	17.18	115.40	6.72	7.09	984.40	4.35	0.13	0.08
10.77	13.71	80.05	6.35	7.01	697.00	3.25	0.20	0.11
10.03	11.57	64.39	6.05	6.77	719.10	3.73	0.20	0.09
10.49	15.35	94.08	5.97	7.12	764.90	3.49	0.35	0.20
10.90	13.39	81.39	6.37	7.12	1007.00	4.27	0.06	0.08
10.77	11.26	63.95	6.11	7.25	761.60	2.91	0.15	0.07
11.73	14.98	93.03	6.45	7.49	1065.00	4.61	0.06	0.06
11.50	15.71	97.01	6.67	7.49	702.00	5.60	0.02	0.07
10.93	16.21	101.25	6.16	6.59	722.20	4.67	0.24	0.14
10.83	11.98	69.12	5.95	6.91	717.20	2.51	0.20	0.09
12.00	13.78	85.95	7.04	7.52	950.20	3.49	0.16	0.09

11.63	13.49	81.02	6.43	7.17	973.80	3.68	0.11	0.05
11.31	13.69	79.46	6.40	7.39	893.10	3.92	0.00	0.01
11.66	13.08	72.06	6.21	7.47	940.60	4.69	0.00	0.00
11.06	15.29	92.82	6.45	7.55	693.90	3.44	0.26	0.11
11.29	14.42	85.28	6.43	7.17	775.20	4.35	0.05	0.03
11.18	15.59	105.27	6.80	7.33	724.60	5.87	0.15	0.14
11.58	15.70	94.23	6.51	7.31	729.70	4.24	0.10	0.04
11.55	15.76	99.81	6.24	7.01	965.60	3.92	0.15	0.11
10.49	10.12	55.53	6.13	7.41	892.10	2.51	0.06	0.06
11.36	12.51	78.43	6.61	7.31	1012.00	2.88	0.13	0.13
12.49	20.80	145.93	6.67	7.31	866.50	4.61	0.24	0.18
11.56	14.77	92.17	6.48	7.55	920.50	3.31	0.10	0.08
11.44	13.13	76.72	6.24	7.17	851.00	3.52	0.07	0.03
11.21	15.29				960.00	4.43	0.21	0.11
11.79	14.49	91.96	6.27	6.96	953.90	2.59	0.11	0.06
10.80	13.41	75.57	6.00	6.69	848.10	3.92	0.09	0.05
12.00	18.37	129.66	6.21	6.83	986.30	5.81	0.07	0.15
12.30	13.91	89.22	7.01	7.12	1186.00	3.07	0.04	0.02
11.56	15.70	106.48	5.97	6.59	1141.00	3.33	0.24	0.16
10.98	11.10	67.34	5.92	6.61	1138.00	2.29	0.13	0.08
10.97	14.11	82.37	6.11	6.59	1136.00	4.24	0.00	0.03
11.67	12.82	73.27	6.29	7.23	1033.00	3.65	0.06	0.09
11.18	11.11	63.30	5.95	6.96	824.30	3.73	0.12	0.10
10.96	13.05	80.74	6.24	7.23	1046.00	2.67	0.20	0.16
10.70	13.02	71.71	6.24	7.36	722.40	4.03	0.10	0.06
11.09	15.15	83.96	6.08	6.91	777.20	4.45	0.04	0.06
11.13	13.02	72.44	5.87	6.72	1007.00	2.69	0.09	0.03
10.85	12.47	70.90	6.11	7.04	878.30	2.85	0.13	0.11
10.78	12.63	75.96	6.29	7.39	914.00	2.69	0.25	0.16
12.16	14.10	87.16	6.32	7.39	1045.00	2.99	0.04	0.02
11.44	14.20	85.80	6.29	7.07	943.50	3.89	0.13	0.10
11.37	12.69	76.62	6.48	7.76	1073.00	2.83	0.15	0.05
10.80	11.90	66.76	6.37	7.09	1013.00	2.83	0.04	0.05
11.52	13.84	96.43	6.56	7.15	1474.00	3.36	0.23	0.18
11.99	16.70	106.55	6.24	6.93	1213.00	4.13	0.07	0.05
10.56	11.40	63.73	5.84	6.64	1288.00	4.11	0.07	0.06
11.39	14.07	85.37	6.32	6.85	1260.00	4.40	0.12	0.05
10.85	12.95	74.12	6.32	7.20	1228.00	3.39	0.02	0.02
10.73	9.01	45.26	6.43	7.07	1378.00	3.52	0.00	0.03
11.15	13.26	77.67	6.61	8.19	491.50	4.77	0.16	0.05
9.74	12.77	70.00	6.11	6.83	294.40	4.27	0.28	0.09
11.36	21.26	136.32	6.48	7.07	241.70	4.48	0.36	0.12
12.16	20.45	134.70	6.61	7.31	579.70	5.49	0.00	0.00
11.26	16.03	105.49	6.56	7.36	480.10	5.81	0.35	0.19
11.65	19.48	124.17	6.48	7.31	323.20	5.41	0.19	0.07
11.21	16.00	97.30	6.40	7.47	270.10	4.96	0.33	0.17
11.28	17.21	109.05	6.19	7.07	666.40	4.05	0.24	0.10

10.67	15.40	88.31	6.05	6.91	271.30	4.27	0.22	0.12
11.46	15.31	91.59	6.64	7.57	368.20	5.09	0.14	0.06
11.10	14.55	93.38	7.28	7.84	573.40	2.77	0.23	0.09
11.95	17.52	112.11	6.59	7.52	563.20	3.87	0.12	0.09
11.65	19.46	138.39	6.72	7.31	647.90	2.61	0.35	0.17
12.00	15.03	90.19	6.88	7.81	639.00	3.92	0.08	0.05
11.25	19.05	123.23	6.61	7.28	506.00	4.03	0.38	0.18
11.36	19.06	123.31	6.61	7.25	559.70	4.03	0.38	0.17
11.39	18.39	113.69	6.32	7.07	478.50	4.08	0.25	0.12
11.15	13.83	73.85	6.29	7.49	594.10	2.99	0.06	0.01
11.36	16.41	98.57	6.43	7.28	647.10	4.48	0.09	0.04
11.42	19.25	125.64	6.80	7.20	530.30	5.57	0.22	0.13
11.12	17.16	106.73	6.40	6.85	382.10	1.63	0.30	0.14
11.02	14.32	85.86	6.27	6.99	557.80	4.40	0.23	0.14

IT750	Iarea	Icurv	IT200	row	CLU3_4	ZACDmm	ZACWmm	ZACA
0.59	1.60	0.09	0.56	222.00	1	1.27372	1.49252	1.67666
0.55	1.47	0.10	0.42	203.00	1	-0.87898	-0.90689	-0.83394
0.41	1.72	0.14	0.48	235.00	1	-0.21548	0.79525	-0.07256
0.43	1.35	0.15	0.51	225.00	1	-1.17018	-0.37369	-0.69338
0.45	1.18	0.16	0.55	201.00	1	-1.36555	-0.88638	-1.14630
0.51	1.40	0.17	0.42	204.00	1	0.25635	-1.31704	0.16952
0.44	1.05	0.18	0.42	217.00	1	-1.26971	-1.97329	-1.25173
0.55	1.91	0.19	0.52	208.00	1	-0.23391	0.71322	-0.22484
0.47	1.38	0.19	0.48	210.00	1	0.39274	-0.43521	0.41550
0.49	1.42	0.20	0.46	214.00	1	-0.74259	0.13901	-0.52939
0.50	1.44	0.20	0.56	234.00	1	-0.64675	0.97982	0.18904
0.49	1.21	0.22	0.51	230.00	1	-1.84106	-0.74283	-1.29468
0.50	1.13	0.22	0.37	228.00	1	-1.99588	-0.57876	-1.33372
0.47	1.38	0.24	0.44	202.00	1	-2.05117	-0.18912	-1.68122
0.44	1.16	0.25	0.32	213.00	1	-0.06803	-0.12759	-0.01400
0.54	1.31	0.26	0.56	212.00	1	-0.77208	-0.92740	-0.70119
0.60	1.65	0.26	0.53	196.00	1	-0.18230	-1.17349	-0.53330
0.44	1.39	0.28	0.38	236.00	1	0.67288	0.89779	1.02851
0.41	1.37	0.28	0.39	243.00	1	-0.26340	0.83627	0.10704
0.44	1.15	0.29	0.39	221.00	1	-1.47982	-1.13247	-1.40400
0.47	1.49	0.30	0.47	207.00	1	0.00201	-0.20963	-0.06476
0.56	1.71	0.30	0.59	244.00	1	0.73923	-0.00455	0.63025
0.54	1.56	0.30	0.48	220.00	1	0.28584	1.08236	0.29446
0.47	1.78	0.32	0.48	247.00	1	0.42223	1.28744	0.21247
0.44	1.21	0.32	0.47	200.00	1	-1.67518	-1.89126	-1.68122
0.55	1.50	0.33	0.50	245.00	1	1.03412	-0.49673	0.82547
0.54	1.30	0.36	0.40	233.00	1	-1.14807	-0.39419	-0.94327
0.47	1.55	0.37	0.50	198.00	1	-1.22548	-1.07095	-1.28687
0.58	1.79	0.37	0.54	248.00	1	0.59179	1.10287	0.45454
0.54	1.35	0.39	0.44	232.00	1	-1.21073	-0.06607	-0.78319
0.42	1.63	0.39	0.52	211.00	1	-0.41821	-1.25552	-1.02526
0.40	1.46	0.40	0.38	215.00	1	-0.48088	-0.47623	-0.54501
0.68	1.93	0.40	0.55	224.00	1	-1.57934	-1.11197	-1.82569
0.36	1.07	0.41	0.35	205.00	1	-0.76840	-0.20963	-0.57234
0.24	1.43	0.41	0.31	242.00	1	-0.88267	-0.88638	-1.36105
0.61	1.74	0.43	0.43	199.00	1	-1.16281	-1.35806	-1.59923
0.43	1.87	0.44	0.40	216.00	1	1.41380	-0.84536	-0.68948
0.34	1.64	0.46	0.40	246.00	1	-0.14175	1.14388	-0.22094
0.65	1.54	0.47	0.44	197.00	1	-0.84212	-1.74770	-1.21268
0.43	1.47	0.50	0.45	227.00	1	-1.27340	-0.08658	-1.13459
0.49	1.75	0.56	0.39	238.00	1	-2.37186	-0.31216	-2.44651
0.63	2.57	0.41	1.24	239.00	2	-1.12964	0.20053	-1.41572
0.43	1.28	0.15	0.40	226.00	2	-0.85318	1.38998	-0.24436
0.35	1.32	0.15	0.30	218.00	2	-1.32869	1.20541	-0.56453
0.52	1.48	0.25	0.43	229.00	2	-1.14069	-0.00455	-0.74804
0.44	1.42	0.28	0.56	195.00	2	-0.77208	1.80013	-0.06866

0.56	1.54	0.31	0.47	237.00	2	-0.86055	1.16439	-0.38102
0.59	1.69	0.35	0.43	240.00	2	-1.19230	-0.20963	-1.15021
0.50	1.46	0.35	0.40	241.00	2	-1.01168	-0.02506	-0.96279
0.55	1.79	0.49	0.47	206.00	2	-1.19230	1.00033	-0.88861
0.60	1.57	0.51	0.46	219.00	2	-0.42190	-1.60415	-0.70509
0.35	1.39	0.26	0.35	209.00	2	-1.94059	-0.53775	-1.17364
0.36	1.39	0.32	0.34	231.00	2	-1.03011	1.55404	-0.49035
0.44	1.22	0.41	0.29	223.00	2	-1.78577	0.34408	-0.97451
0.66	1.78	0.52	0.50	166.00	1	-0.11595	0.20053	-1.05650
0.62	1.04	0.05	0.39	190.00	1	0.61759	-1.02993	0.85281
0.39	1.77	0.07	0.50	187.00	1	-0.04591	0.46713	0.00162
0.58	1.42	0.08	0.39	153.00	1	-0.60621	-0.92740	-0.62310
0.57	1.63	0.09	0.49	146.00	1	-0.99325	0.09799	-0.84175
0.54	1.38	0.18	0.50	154.00	1	0.58442	-1.52212	0.23589
0.46	1.42	0.23	0.39	169.00	1	0.54387	0.50814	0.92309
0.40	1.47	0.23	0.44	184.00	1	0.33376	1.16439	0.31789
0.54	1.27	0.25	0.39	173.00	1	0.85719	1.43099	0.86842
0.44	1.44	0.27	0.44	180.00	1	0.00201	1.32845	0.56778
0.53	1.49	0.29	0.45	183.00	1	0.59179	0.61068	1.09879
0.47	1.34	0.30	0.48	152.00	1	-1.33606	-0.49673	-0.97451
0.70	1.83	0.30	0.58	189.00	1	0.61759	0.24154	0.66539
0.48	1.29	0.30	0.47	149.00	1	-1.18861	-1.46060	-1.13850
0.63	1.56	0.31	0.50	151.00	1	-1.12964	-1.02993	-1.25563
0.35	1.40	0.31	0.36	148.00	1	-1.11489	0.48764	-0.83785
0.43	1.19	0.32	0.43	150.00	1	-1.34343	-1.66567	-1.31420
0.49	1.84	0.35	0.44	188.00	1	1.21474	2.23079	1.11050
0.39	0.94	0.35	0.27	143.00	1	-1.14069	-0.37369	-0.86128
0.47	1.36	0.36	0.36	159.00	1	0.44803	0.15951	0.39598
0.42	1.30	0.36	0.40	160.00	1	0.07573	-0.02506	-0.08818
0.50	1.56	0.37	0.43	193.00	1	-0.49562	-1.11197	-0.80271
0.36	1.44	0.37	0.43	179.00	1	-0.55829	-1.35806	-1.07993
0.43	1.54	0.38	0.48	191.00	1	-1.26234	-0.43521	-1.25954
0.44	1.24	0.38	0.35	171.00	1	-0.41084	0.91830	-0.25998
0.50	1.64	0.39	0.50	155.00	1	0.02781	1.08236	0.56387
0.51	1.42	0.39	0.44	156.00	1	-0.05697	-0.25064	-0.02961
0.52	1.91	0.40	0.51	185.00	1	0.61759	0.97982	0.29446
0.40	1.46	0.40	0.38	161.00	1	-0.48088	-0.47623	-0.54501
0.52	1.32	0.40	0.46	174.00	1	0.75398	-0.27115	0.91528
0.50	1.62	0.41	0.54	178.00	1	-0.32606	-0.10709	-0.34198
0.54	1.52	0.41	0.43	167.00	1	-0.92690	0.22104	-0.70509
0.51	1.67	0.42	0.45	181.00	1	0.77609	0.54916	0.69272
0.54	1.77	0.43	0.45	192.00	1	-0.13069	0.83627	-0.34978
0.61	1.81	0.44	0.51	177.00	1	-1.01905	-1.21450	-1.43133
0.50	1.65	0.45	0.42	194.00	1	-0.10489	-0.23013	-0.61139
0.59	1.58	0.45	0.43	182.00	1	0.81296	-0.27115	0.57558
0.55	1.46	0.46	0.39	163.00	1	-0.69467	-2.01430	-1.36886
0.52	1.73	0.50	0.40	142.00	1	-0.33343	0.61068	-0.33417

0.43	1.13	0.50	0.30	175.00	1	-0.91215	-1.85024	-1.12288
0.63	1.70	0.50	0.61	164.00	1	-0.64307	-0.76333	-0.81442
0.39	1.38	0.51	0.38	147.00	1	1.24055	1.20541	1.32916
0.44	1.77	0.53	0.40	162.00	1	-0.34818	-0.86587	-0.86128
0.40	1.58	0.53	0.45	176.00	1	-1.21442	-1.46060	-1.73589
0.50	1.62	0.54	0.58	186.00	1	-0.32606	0.69271	0.41550
0.74	2.01	0.57	0.57	170.00	1	-0.97482	0.05698	-1.28296
0.39	1.27	0.12	0.32	157.00	2	-1.25865	-0.90689	-0.82223
0.47	1.47	0.21	0.63	145.00	2	-0.68730	1.63607	0.30227
0.67	1.72	0.46	0.47	144.00	2	-0.91953	0.87728	-0.89642
0.35	1.88	0.70	0.40	165.00	2	-0.55091	1.20541	-0.70509
0.60	1.44	0.27	0.54	172.00	3	-1.07434	-2.34243	-1.13069
0.62	1.88	0.28	0.52	168.00	3	1.36219	1.41048	1.35649
0.54	1.72	0.35	0.43	158.00	3	0.40011	-0.66080	-0.09599
0.45	1.73	0.08	0.40	128.00	1	0.77241	0.79525	0.76691
0.43	1.60	0.15	0.42	100.00	1	2.14365	0.28256	2.19205
0.47	0.99	0.16	0.31	137.00	1	-0.74628	-1.05044	-0.51377
0.48	1.30	0.19	0.38	132.00	1	-0.38872	-1.25552	-0.13504
0.46	1.20	0.19	0.34	107.00	1	0.48489	-0.16861	1.00899
0.40	1.00	0.21	0.39	104.00	1	-0.61726	-0.70181	-0.25608
0.51	1.39	0.24	0.42	102.00	1	0.26372	0.36459	0.66148
0.35	1.08	0.24	0.32	133.00	1	-0.25971	-1.48110	-0.51377
0.49	1.61	0.25	0.45	94.00	1	0.54755	0.26205	0.66539
0.49	1.36	0.26	0.28	136.00	1	-0.22285	-0.31216	-0.18970
0.58	1.70	0.27	0.44	106.00	1	0.68763	0.71322	1.11831
0.48	1.29	0.28	0.40	105.00	1	0.05730	-0.16861	0.43112
0.41	1.50	0.28	0.43	135.00	1	-0.66150	0.36459	-0.62700
0.51	1.52	0.28	0.44	121.00	1	-0.91584	1.06185	-0.33417
0.38	1.67	0.29	0.51	134.00	1	1.15577	1.94369	0.92309
0.44	1.29	0.30	0.39	140.00	1	0.38536	1.08236	0.86842
0.50	1.20	0.32	0.51	103.00	1	-0.41084	-1.46060	-0.45130
0.26	1.40	0.32	0.43	112.00	1	-0.46613	-0.41470	-0.71290
0.59	1.40	0.32	0.47	139.00	1	-1.80420	-1.31704	-1.63437
0.42	1.42	0.33	0.39	122.00	1	0.28952	1.10287	0.25932
0.46	1.56	0.37	0.46	138.00	1	-1.40610	-1.62466	-1.59923
0.48	1.68	0.38	0.67	89.00	1	1.41380	2.02572	1.61028
0.55	1.60	0.40	0.50	108.00	1	-0.38504	-1.23501	-0.78319
0.34	1.37	0.41	0.30	109.00	1	0.43697	0.30307	0.15390
0.58	1.53	0.42	0.46	110.00	1	-0.72048	-1.19400	-1.01355
0.43	1.29	0.42	0.37	113.00	1	-0.06803	-0.04556	0.04848
0.39	1.75	0.49	0.46	90.00	1	-0.13807	0.09799	-0.58796
0.49	2.13	0.57	0.43	123.00	1	0.86088	-0.23013	-0.25608
0.32	1.70	0.62	0.36	111.00	1	-0.62464	-0.02506	-1.19706
0.40	0.93	0.18	0.28	141.00	2	-1.94059	-0.53775	-1.17364
0.31	1.32	0.34	0.40	92.00	2	-1.20336	1.26693	-0.74804
0.45	2.08	0.38	0.50	119.00	2	-0.39241	0.71322	-0.73243
0.44	1.80	0.46	0.39	91.00	2	0.07573	0.75424	-0.27950

0.46	1.79	0.59	0.48	118.00	2	0.16420	0.32358	0.21637
0.39	1.43	0.05	0.38	129.00	3	1.16682	-0.20963	1.00508
0.39	1.57	0.11	0.43	130.00	3	0.76135	0.65170	0.83328
0.60	1.60	0.13	0.44	131.00	3	0.59179	-0.41470	0.41160
0.43	1.74	0.14	0.44	125.00	3	0.78715	-0.27115	0.57558
0.44	1.72	0.16	0.51	124.00	3	1.22949	0.54916	1.11831
0.56	1.70	0.21	0.43	127.00	3	1.28847	0.77475	1.41505
0.48	1.40	0.22	0.57	96.00	3	1.27372	-0.59927	1.25887
0.52	1.44	0.23	0.44	101.00	3	1.00832	-0.41470	0.73567
0.40	1.47	0.23	0.40	120.00	3	1.23686	2.51790	1.87579
0.28	1.15	0.24	0.32	117.00	3	1.50226	0.83627	1.78989
0.46	1.51	0.25	0.47	116.00	3	-0.05329	-1.48110	-0.26779
0.54	1.78	0.30	0.47	95.00	3	1.38431	0.22104	0.74738
0.38	1.61	0.30	0.51	98.00	3	0.66920	0.50814	0.51311
0.59	1.94	0.30	0.56	114.00	3	3.29004	2.35384	3.76557
0.29	1.33	0.31	0.35	93.00	3	0.97883	0.38510	0.86061
0.47	1.69	0.35	0.40	126.00	3	1.27372	0.15951	0.99337
0.44	1.44	0.36	0.37	97.00	3	-0.00905	-1.25552	-0.50596
0.64	1.99	0.37	0.56	115.00	3	1.38431	0.81576	1.26278
0.48	1.85	0.41	0.42	99.00	3	2.06255	0.20053	1.49705
0.44	1.41	0.07	0.43	4.00	1	-0.49562	-0.41470	-0.31464
0.40	1.59	0.11	0.43	39.00	1	-0.47719	-0.59927	-0.54501
0.28	1.71	0.14	0.32	25.00	1	2.28741	0.77475	2.57860
0.44	1.38	0.21	0.44	61.00	1	0.83507	0.50814	0.81766
0.37	1.38	0.22	0.44	76.00	1	-0.19704	-0.92740	-0.40835
0.58	1.15	0.23	0.48	3.00	1	-0.79420	0.05698	-0.20141
0.51	1.63	0.24	0.51	74.00	1	0.25635	0.48764	0.54044
0.43	1.50	0.30	0.43	56.00	1	-0.65413	0.50814	-0.63091
0.43	1.40	0.32	0.50	86.00	1	0.22317	1.10287	0.43502
0.39	1.30	0.32	0.35	72.00	1	0.24161	0.97982	0.80205
0.63	1.97	0.33	0.48	13.00	1	1.64602	-0.47623	1.03241
0.39	1.26	0.33	0.43	17.00	1	0.33007	0.38510	0.52873
0.50	1.67	0.34	0.47	87.00	1	1.16682	1.04135	1.07536
0.46	1.32	0.34	0.33	82.00	1	0.33744	-0.10709	0.15780
0.48	1.40	0.34	0.51	6.00	1	0.49595	0.56967	0.83719
0.35	1.69	0.35	0.46	69.00	1	0.54018	1.53353	0.74738
0.42	1.29	0.35	0.31	41.00	1	0.66182	1.41048	1.24326
0.38	1.33	0.35	0.41	15.00	1	0.21949	-1.05044	-0.11161
0.38	1.04	0.35	0.44	48.00	1	-0.84212	-2.56801	-0.94717
0.36	1.20	0.36	0.41	18.00	1	0.65077	-1.62466	0.52873
0.55	1.33	0.36	0.39	32.00	1	-0.39978	-0.78384	-0.23655
0.36	1.35	0.36	0.36	47.00	1	-0.84580	-1.05044	-1.06821
0.55	1.63	0.36	0.43	64.00	1	0.04624	0.91830	0.38426
0.59	1.35	0.37	0.49	78.00	1	0.35219	0.44662	0.66929
0.47	1.14	0.37	0.42	52.00	1	0.48120	-0.72232	0.86452
0.31	1.37	0.38	0.36	83.00	1	-0.24496	-0.92740	-0.78709
0.33	1.58	0.38	0.44	73.00	1	-0.17124	1.47201	-0.08428

0.38	1.65	0.38	0.43	71.00	1	-0.17124	0.71322	-0.19751
0.55	1.38	0.39	0.40	26.00	1	-0.01642	0.05698	-0.11942
0.48	1.75	0.39	0.52	70.00	1	0.01675	0.77475	-0.35759
0.46	1.49	0.40	0.37	60.00	1	0.76872	-0.45572	0.50530
0.59	1.48	0.40	0.44	5.00	1	0.12365	0.01596	0.16561
0.63	1.26	0.40	0.48	16.00	1	-0.00537	-0.20963	0.62244
0.48	1.75	0.41	0.39	19.00	1	0.83876	0.61068	0.66539
0.37	1.34	0.42	0.35	75.00	1	0.58810	0.54916	0.68882
0.44	1.37	0.43	0.40	24.00	1	-1.12595	-1.62466	-1.51333
0.43	1.89	0.44	0.50	34.00	1	-0.46982	0.15951	-0.58015
0.39	1.41	0.44	0.43	79.00	1	2.00726	2.47689	2.65669
0.51	1.56	0.45	0.36	62.00	1	0.28584	0.56967	0.30227
0.42	1.56	0.45	0.40	63.00	1	-0.23759	0.32358	-0.33807
0.32	1.37	0.45	0.45	40.00	1	0.33376	-0.14810	0.50530
0.32	1.81	0.45	0.40	80.00	1	0.44066	1.04135	0.19294
0.48	1.52	0.45	0.40	22.00	1	0.19000	-0.98892	-0.22874
0.69	1.25	0.46	0.50	8.00	1	1.36588	1.47201	1.70789
0.40	1.68	0.46	0.36	85.00	1	-0.14544	2.08724	-0.03352
0.56	1.92	0.46	0.52	77.00	1	0.47383	0.56967	0.66539
0.34	1.35	0.47	0.26	38.00	1	-1.11858	-0.61978	-1.13069
0.53	1.46	0.47	0.54	65.00	1	0.02781	-0.64029	0.04457
0.45	1.60	0.48	0.44	35.00	1	-0.23022	0.79525	-0.45911
0.43	1.58	0.48	0.43	51.00	1	-0.85318	-0.20963	-1.12678
0.38	1.43	0.48	0.43	81.00	1	-0.38135	-0.66080	-0.36931
0.57	1.61	0.49	0.56	14.00	1	0.02412	-1.19400	-0.38102
0.67	1.90	0.49	0.47	20.00	1	1.14102	-0.39419	0.45064
0.35	1.72	0.51	0.39	27.00	1	0.30058	-0.31216	-0.38102
0.59	1.99	0.52	0.48	21.00	1	0.02412	-0.88638	-0.59577
0.62	2.19	0.52	0.60	23.00	1	-0.24128	-1.02993	-0.53330
0.60	2.06	0.53	0.42	30.00	1	0.39274	1.80013	0.04067
0.38	1.43	0.53	0.37	28.00	1	0.14208	0.32358	0.07971
0.33	1.59	0.54	0.38	66.00	1	-0.51774	0.18002	-0.50987
0.61	1.81	0.59	0.41	29.00	1	-0.45139	-0.98892	-0.81833
0.47	1.52	0.42	0.36	1.00	2	-1.16650	0.48764	-0.06085
0.48	1.60	0.43	0.41	31.00	2	0.82770	1.45150	1.05584
0.43	1.09	0.44	0.30	2.00	2	-0.97482	-1.48110	-1.01355
0.43	1.45	0.45	0.39	36.00	2	-0.08277	0.22104	0.02895
0.52	1.70	0.45	0.47	33.00	2	-0.03485	-0.88638	-0.40835
0.59	1.86	0.66	0.56	37.00	2	-1.83000	-1.13247	-1.94673
0.52	1.45	0.16	0.59	45.00	3	-0.39610	-0.27115	-0.28731
0.52	1.11	0.20	0.40	46.00	3	-0.32606	-3.16274	-0.47863
0.55	1.65	0.21	0.50	44.00	3	3.02464	0.15951	2.83630
0.75	1.72	0.22	0.60	68.00	3	2.15839	1.80013	2.52003
0.43	1.16	0.22	0.60	53.00	3	-0.01274	-0.04556	0.79424
0.62	1.71	0.24	0.63	43.00	3	2.05887	0.75424	2.14129
0.48	1.55	0.27	0.66	50.00	3	0.73555	-0.14810	0.78252
0.34	1.02	0.28	0.30	7.00	3	1.21843	-0.00455	1.25497

0.63	1.72	0.28	0.66	49.00	3	1.18157	-1.25552	0.54825
0.47	1.31	0.30	0.53	54.00	3	0.54755	0.36459	0.51311
0.40	1.56	0.31	0.35	84.00	3	0.23423	-0.37369	0.21637
0.48	1.65	0.34	0.47	42.00	3	1.38799	1.36947	1.37601
0.37	1.34	0.35	0.31	9.00	3	1.87825	0.75424	2.13348
0.51	1.94	0.36	0.48	67.00	3	0.62865	1.47201	0.40379
0.48	1.60	0.36	0.47	11.00	3	1.95566	-0.06607	1.97340
0.51	1.63	0.38	0.44	58.00	3	1.95566	0.15951	1.97730
0.46	1.85	0.39	0.52	57.00	3	2.20631	0.22104	1.71570
0.51	1.82	0.40	0.49	59.00	3	1.07467	-0.27115	-0.06476
0.56	1.58	0.40	0.48	88.00	3	1.12996	0.15951	0.94261
0.58	1.47	0.40	0.62	12.00	3	1.62022	0.28256	2.05149
0.48	2.10	0.40	0.47	10.00	3	2.29847	-0.33267	1.23545
0.44	1.49	0.41	0.48	55.00	3	0.11628	-0.53775	0.12657

ZLENSVAUI	ZPUPILDmr	ZAOD750	ZIT750	ZIarea	ZIcurv
-0.79315	-1.48996	-0.29690	1.17949	0.30330	-2.07179
-0.45296	-0.87776	0.46398	0.74845	-0.19589	-1.94898
0.05364	-0.87776	-0.12299	-0.70630	0.80250	-1.66242
-0.07359	1.11758	1.01835	-0.54466	-0.69509	-1.53960
-0.05497	1.20827	-1.17193	-0.27526	-1.35403	-1.49867
-0.51270	-0.18619	0.02919	0.37129	-0.49541	-1.37586
-0.67872	0.39200	-0.67191	-0.38302	-1.87320	-1.29398
0.14829	0.11991	-0.07951	0.74845	1.54131	-1.21211
-0.51308	0.59607	0.23572	-0.11363	-0.57528	-1.21211
0.71463	0.41467	-0.75887	0.15577	-0.41554	-1.17117
1.12580	0.84549	0.50203	0.20965	-0.33567	-1.17117
0.80772	1.02688	-0.57408	0.15577	-1.25419	-1.00742
0.76506	0.63008	-0.76974	0.20965	-1.57368	-0.96648
0.23945	0.44869	-1.17193	-0.11363	-0.57528	-0.80273
-0.17367	0.35799	-0.59582	-0.38302	-1.43390	-0.72086
0.09010	0.48270	-1.17193	0.64069	-0.85483	-0.67992
-0.36103	0.11991	-0.95996	1.34112	0.50298	-0.63898
-0.01967	0.48270	0.42594	-0.38302	-0.53535	-0.51617
0.63705	1.32164	-0.56865	-0.70630	-0.59525	-0.47524
0.14519	-0.12951	-1.17193	-0.38302	-1.49381	-0.43430
-0.53675	0.32398	-0.12299	-0.05975	-0.13599	-0.35242
-0.59804	0.59607	-0.35669	0.91009	0.76256	-0.35242
-0.51658	0.57339	-0.97083	0.64069	0.16353	-0.31149
-0.38934	-0.63968	-1.17193	-0.11363	1.04211	-0.18867
0.22548	0.21061	-0.92192	-0.38302	-1.23422	-0.14774
-0.20703	0.63008	-0.03059	0.74845	-0.09606	-0.10680
0.52068	0.77746	-1.17193	0.64069	-0.89477	0.17976
-0.13488	-0.69637	-0.31321	-0.11363	0.10362	0.22070
-0.16242	-0.54898	-0.95453	1.07173	1.06208	0.22070
0.60602	0.48270	-0.95453	0.64069	-0.67512	0.38445
-0.78927	1.25362	-0.49256	-0.65242	0.42311	0.42538
0.12463	-0.96846	0.06724	-0.81406	-0.23583	0.46632
0.14092	-0.76439	-1.17193	2.20320	1.62118	0.46632
0.70299	1.11758	-0.74257	-1.24510	-1.79333	0.54820
-0.50339	1.25362	-0.30234	-2.53821	-0.35564	0.58913
-0.58407	0.82281	-1.17193	1.44888	0.86240	0.71195
0.11066	0.46002	-0.15560	-0.54466	1.40154	0.79382
1.03659	-1.03648	0.22485	-1.51449	0.46305	0.95757
-0.57399	-0.73038	-1.17193	1.87992	0.08366	1.08038
1.15296	-0.24288	-0.94909	-0.54466	-0.19589	1.32600
0.88918	-0.49230	-1.17193	0.15577	0.90234	1.81725
1.43613	1.01554	-1.17193	1.61052	4.17707	0.58913
1.93652	1.71844	-0.97083	-0.49078	-0.95467	-1.53960
2.38649	-0.24288	0.03463	-1.40674	-0.81490	-1.53960
1.50207	0.72078	-1.17193	0.47905	-0.15596	-0.72086
1.57189	1.50304	-0.73713	-0.38302	-0.41554	-0.51617

1.41285	0.21061	-1.17193	0.91009	0.08366	-0.22961
1.29260	-0.60567	-0.97083	1.17949	0.68269	0.05695
1.54086	-0.09550	-1.17193	0.20965	-0.23583	0.09789
1.96367	-0.27689	-0.98170	0.74845	1.06208	1.24413
1.38570	-0.15218	-1.17193	1.34112	0.20346	1.36694
2.80542	-0.76439	0.45311	-1.40674	-0.51538	-0.67992
2.95283	4.07657	-0.95453	-1.24510	-0.51538	-0.14774
3.11187	0.44869	-0.59582	-0.38302	-1.21426	0.58913
0.58662	-1.96612	-0.41104	1.93380	1.02215	1.48975
-0.49330	2.26263	-0.33495	1.50276	-1.93310	-2.39929
-0.41145	-1.14985	1.00748	-0.97570	0.98221	-2.19460
-0.37848	-0.58299	-0.35125	1.07173	-0.41554	-2.11273
0.74566	0.81147	-0.95453	1.01785	0.44308	-2.03085
-0.72915	0.30130	-0.11755	0.64069	-0.55532	-1.33492
0.97064	-0.12951	0.58355	-0.22138	-0.41554	-0.88461
-0.37499	-0.12951	-0.95453	-0.81406	-0.19589	-0.88461
-0.24039	1.48036	-0.35125	0.64069	-1.01458	-0.72086
0.59050	0.81147	1.65423	-0.38302	-0.31570	-0.55711
0.11377	1.32164	0.56725	0.58681	-0.11602	-0.39336
0.99004	0.84549	-1.17193	-0.11363	-0.71506	-0.35242
0.45861	0.08590	-0.37843	2.36484	1.24179	-0.31149
-0.44637	0.50537	-0.33495	0.04801	-0.93471	-0.31149
-0.47003	0.48270	-1.17193	1.61052	0.14356	-0.27055
0.64868	0.14258	-1.17193	-1.35286	-0.47544	-0.22961
0.18281	0.32398	-0.76431	-0.54466	-1.33406	-0.14774
0.59050	0.05189	-0.95996	0.15577	1.28173	0.05695
0.84651	0.44869	-0.08494	-0.92182	-2.31249	0.09789
0.38685	0.11991	-0.78061	-0.11363	-0.63519	0.13882
0.34108	-0.51497	-0.75344	-0.65242	-0.89477	0.17976
0.26660	-0.36759	-0.34582	0.20965	0.16353	0.22070
0.11415	-0.87776	-0.56322	-1.24510	-0.33567	0.26164
1.15296	-0.49230	-0.36212	-0.54466	0.06369	0.34351
0.59826	0.11991	-1.17193	-0.38302	-1.11442	0.34351
0.68360	0.66409	0.42050	0.20965	0.48301	0.38445
-0.35521	0.77746	-0.30234	0.37129	-0.39557	0.38445
-0.04372	-1.27456	-0.56322	0.47905	1.54131	0.46632
0.12463	-0.87776	0.06724	-0.81406	-0.23583	0.46632
0.17467	1.11758	0.00202	0.47905	-0.79493	0.50726
0.68747	0.59607	-1.17193	0.20965	0.40314	0.58913
1.09089	1.04955	-1.17193	0.64069	-0.01618	0.58913
-0.72876	0.86816	-0.51974	0.37129	0.60282	0.67101
0.44310	-0.15218	-0.78605	0.64069	0.98221	0.71195
-0.29004	-0.63968	-0.78605	1.44888	1.16192	0.83476
0.55947	-1.03648	-0.97627	0.20965	0.52295	0.91663
0.03308	0.44869	-1.17193	1.23336	0.24340	0.91663
-0.46266	-1.42194	-0.93822	0.74845	-0.25580	0.95757
0.81936	-0.73038	-0.57952	0.47905	0.84244	1.32600

0.97064	-0.51497	-1.17193	-0.54466	-1.57368	1.32600
0.96676	0.72078	-0.93822	1.61052	0.72263	1.32600
0.25302	-0.94578	-0.62843	-0.92182	-0.55532	1.40788
0.81548	-2.60100	-0.35669	-0.38302	0.98221	1.57163
-0.31331	-1.42194	-0.92735	-0.81406	0.24340	1.57163
0.49352	0.39200	0.03463	0.20965	0.40314	1.61256
0.97452	-0.00480	-0.97083	2.79587	1.94067	1.89913
1.66887	-0.63968	0.64334	-0.97570	-1.01458	-1.78523
1.30036	1.75246	-0.30234	-0.05975	-0.19589	-1.04836
1.63396	0.53938	-1.17193	2.09544	0.80250	0.99851
2.12659	0.68677	-0.95996	-1.35286	1.42150	2.92256
-1.24040	-0.03881	-0.70452	1.34112	-0.31570	-0.59805
-1.09959	0.35799	-0.70996	1.50276	1.44147	-0.47524
-0.87034	-0.67369	0.27920	0.64069	0.80250	0.05695
-0.54140	-1.21787	0.45311	-0.27526	0.82247	-2.11273
-0.27608	-0.40160	2.55643	-0.49078	0.30330	-1.53960
-0.03713	0.66409	-0.56322	-0.11363	-2.13278	-1.45773
0.03076	-0.51497	0.62160	0.04801	-0.89477	-1.21211
0.06373	0.77746	1.14335	-0.22138	-1.27416	-1.21211
-0.12557	0.63008	0.77377	-0.81406	-2.09284	-1.08929
-0.39594	0.75479	0.22485	0.31741	-0.53535	-0.84367
-0.63993	-0.24288	0.27376	-1.35286	-1.77336	-0.80273
-0.41494	0.53938	0.77377	0.15577	0.34324	-0.72086
-0.07320	-0.36759	-0.73713	0.15577	-0.65516	-0.63898
0.10523	0.02921	0.59442	1.07173	0.70266	-0.59805
-0.25746	0.99287	0.09441	0.04801	-0.93471	-0.51617
-0.08057	-1.42194	-0.36212	-0.70630	-0.09606	-0.47524
0.39461	1.04955	-0.68278	0.31741	0.00378	-0.47524
-0.03208	-1.03648	-0.39473	-1.08346	0.58285	-0.43430
0.27513	0.68677	-0.32951	-0.38302	-0.93471	-0.35242
-0.27957	0.44869	-0.72626	0.20965	-1.29413	-0.18867
0.47025	-1.63735	0.92595	-2.37657	-0.47544	-0.14774
0.18204	0.17659	-1.17193	1.23336	-0.47544	-0.14774
0.23130	0.86816	-0.50887	-0.65242	-0.39557	-0.10680
0.39965	0.67543	-0.69909	-0.22138	0.16353	0.26164
0.26582	2.22862	-0.23712	0.04801	0.64276	0.30257
-0.24892	-0.94578	-0.43821	0.74845	0.30330	0.46632
-0.02976	-0.78706	0.40963	-1.51449	-0.59525	0.58913
0.19600	-0.63968	-0.80235	1.07173	0.02375	0.63007
0.67972	-0.42427	0.01289	-0.54466	-0.93471	0.67101
-0.73264	-1.14985	0.24115	-0.97570	0.92231	1.20319
-0.59183	-3.30391	0.14332	0.15577	2.43987	1.85819
0.90470	0.68677	-0.10668	-1.67613	0.72263	2.26756
1.57189	-0.36759	-0.06864	-0.81406	-2.35243	-1.29398
2.16538	1.17426	-0.97627	-1.83777	-0.79493	0.01601
1.45940	-0.84375	-0.53604	-0.27526	2.24019	0.30257
1.31200	-0.69637	-0.97083	-0.38302	1.12199	0.99851

1.82015	-0.94578	1.01835	-0.22138	1.06208	2.06287
-1.22915	-1.33124	1.47488	-0.92182	-0.35564	-2.35835
-1.27570	-0.58299	1.32814	-0.97570	0.18350	-1.90804
-1.18765	0.50537	-0.55778	1.34112	0.30330	-1.70335
-1.18959	-0.78706	1.33901	-0.54466	0.86240	-1.66242
-1.18959	-0.18619	1.07813	-0.38302	0.80250	-1.49867
-1.09533	-0.06149	0.34985	0.91009	0.70266	-1.08929
-1.78269	0.75479	2.44773	0.04801	-0.49541	-0.96648
-1.06662	-0.49230	-0.24799	0.47905	-0.33567	-0.92555
-1.50689	1.20827	0.61073	-0.81406	-0.21586	-0.88461
-0.93241	0.86816	2.01837	-2.10717	-1.49381	-0.80273
-1.69308	-0.73038	1.04009	-0.22138	-0.03615	-0.76180
-2.09534	-0.73038	-0.48713	0.64069	1.04211	-0.31149
-1.01076	0.32398	0.29550	-1.08346	0.36321	-0.31149
-1.85717	0.86816	1.11617	1.17949	1.68109	-0.31149
-1.28036	-0.22021	1.38792	-1.99941	-0.75500	-0.22961
-1.02783	-0.58299	0.43681	-0.11363	0.66272	0.09789
-1.58525	-1.51264	0.56725	-0.38302	-0.31570	0.13882
-1.39673	0.81147	0.12158	1.77216	1.86080	0.22070
-1.17562	-0.58299	1.75206	0.04801	1.32166	0.54820
0.02455	-0.45829	0.33355	-0.38302	-0.45548	-2.19460
-0.45102	-0.33358	0.76290	-0.81406	0.26337	-1.90804
-0.09648	-2.72571	4.40430	-2.10717	0.76256	-1.66242
-0.26715	0.39200	0.95856	-0.38302	-0.57528	-1.04836
-0.78772	-0.78706	1.04552	-1.13734	-0.55532	-1.00742
0.68747	1.95652	-1.17193	1.07173	-1.47384	-0.92555
-0.57554	0.41467	-0.11212	0.37129	0.42311	-0.84367
0.12075	-1.42194	-0.75344	-0.54466	-0.09606	-0.35242
0.17622	1.38967	0.32811	-0.54466	-0.47544	-0.18867
-0.16164	0.93618	1.08900	-0.97570	-0.87480	-0.14774
-0.82030	-1.30857	0.34985	1.61052	1.80089	-0.10680
-0.56661	1.04955	0.09984	-0.97570	-1.03455	-0.06586
-0.58640	0.39200	0.04006	0.20965	0.60282	-0.02493
-0.45141	0.41467	-0.53604	-0.22138	-0.79493	-0.02493
-0.46304	1.57106	0.26833	0.04801	-0.49541	0.01601
0.11221	-0.40160	0.90421	-1.40674	0.66272	0.05695
0.37095	0.39200	0.25202	-0.65242	-0.93471	0.05695
-0.74389	-0.85509	0.98574	-1.08346	-0.75500	0.09789
-0.65816	-0.31090	0.95856	-1.08346	-1.93310	0.09789
-0.48050	-0.58299	2.64882	-1.24510	-1.27416	0.13882
0.45861	0.30130	-0.47626	0.74845	-0.77496	0.13882
-0.49330	-1.24055	0.49116	-1.24510	-0.69509	0.17976
0.68360	0.68677	-0.47626	0.80233	0.42311	0.17976
-0.72449	1.80914	-0.96540	1.23336	-0.69509	0.22070
-0.64613	0.75479	1.42596	-0.05975	-1.51377	0.26164
-0.66553	-1.69403	1.01835	-1.83777	-0.59525	0.30257
0.23828	-0.58299	0.51290	-1.56837	0.24340	0.30257

0.32983	-0.36759	0.04006	-1.08346	0.52295	0.30257
0.01679	-0.09550	-1.17193	0.80233	-0.55532	0.38445
0.20104	0.77746	-1.17193	0.04801	0.90234	0.42538
-0.75591	-0.63968	1.61075	-0.22138	-0.13599	0.46632
-0.44055	0.39200	-0.57952	1.17949	-0.17593	0.50726
-0.63683	2.11524	0.49116	1.66440	-1.03455	0.50726
-0.61704	0.26729	-0.12842	0.04801	0.92231	0.58913
0.29802	-0.09550	0.43681	-1.13734	-0.73503	0.63007
0.01291	-1.69403	-0.52517	-0.38302	-0.61522	0.75288
0.47801	-1.27456	0.26289	-0.54466	1.46144	0.83476
-0.08639	0.68677	1.44227	-0.97570	-0.45548	0.83476
0.12308	-0.78706	-0.07951	0.31741	0.14356	0.87569
-0.14652	-0.54898	-0.36756	-0.65242	0.16353	0.87569
0.27630	0.48270	1.07269	-1.73001	-0.59525	0.91663
0.25264	-1.60334	0.05637	-1.67613	1.16192	0.91663
-0.15777	-0.09550	-0.16103	0.04801	0.00378	0.91663
0.37832	2.04722	-0.40017	2.31096	-1.09445	0.95757
1.15296	-1.05915	-0.75344	-0.81406	0.62279	0.99851
0.97840	-0.76439	1.44770	0.91009	1.58125	0.99851
0.96676	-1.94345	0.24659	-1.51449	-0.69509	1.03944
0.95901	0.26729	-1.17193	0.58681	-0.23583	1.08038
0.55947	-0.40160	-0.53604	-0.27526	0.30330	1.12132
-0.25009	-0.31090	0.12702	-0.54466	0.24340	1.12132
0.60989	-1.51264	0.95856	-1.08346	-0.35564	1.16226
-0.64536	0.02921	-0.09581	1.01785	0.36321	1.20319
-0.43279	0.50537	-0.77518	2.04156	1.50138	1.24413
0.45861	-1.48996	-0.19364	-1.40674	0.78253	1.40788
-0.04062	-1.30857	0.22485	1.23336	1.88077	1.44882
0.09786	-1.48996	1.57814	1.50276	2.67948	1.48975
0.60602	-1.14985	-0.77518	1.34112	2.16032	1.57163
0.21229	-0.12951	0.26833	-1.08346	-0.37561	1.57163
0.71463	-1.33124	0.49116	-1.56837	0.28333	1.61256
0.48189	-1.33124	-0.79148	1.44888	1.14195	2.06287
2.27012	-0.73038	1.33357	-0.11363	-0.01618	0.67101
1.25769	0.14258	-0.37299	0.04801	0.32327	0.75288
1.54862	0.11991	-0.42734	-0.54466	-1.73342	0.83476
1.44001	0.44869	0.13789	-0.54466	-0.27577	0.87569
1.31588	-0.69637	-0.98714	0.47905	0.70266	0.91663
1.89773	-0.54898	-1.17193	1.17949	1.36160	2.63600
-1.54103	0.86816	0.52377	0.47905	-0.29573	-1.49867
-2.30558	0.30130	1.91511	0.47905	-1.65355	-1.13023
-2.51001	0.53938	2.70317	0.74845	0.52295	-1.08929
-1.19890	1.68443	-1.17193	2.90363	0.78253	-0.96648
-1.58525	2.04722	2.59991	-0.54466	-1.45387	-0.96648
-2.19387	1.59374	0.83899	1.50276	0.76256	-0.80273
-2.39984	1.08357	2.43142	0.04801	0.12359	-0.55711
-0.86258	0.05189	1.46401	-1.51449	-1.99300	-0.47524

-2.39519	0.30130	1.18683	1.61052	0.78253	-0.47524
-2.01931	1.23095	0.31181	-0.05975	-0.85483	-0.35242
-1.22333	-1.39927	1.33901	-0.81406	0.14356	-0.27055
-1.26290	-0.15218	0.07811	0.04801	0.50298	0.01601
-0.93435	-1.58066	2.63795	-1.13734	-0.73503	0.05695
-0.96887	-0.09550	-0.27516	0.31741	1.68109	0.13882
-1.48478	0.02921	3.00753	0.04801	0.32327	0.17976
-1.27648	0.02921	2.99122	0.37129	0.42311	0.30257
-1.59145	0.08590	1.58901	-0.22138	1.32166	0.42538
-1.14304	-1.14985	-0.51974	0.31741	1.18189	0.46632
-0.93745	0.53938	-0.21538	0.91009	0.24340	0.46632
-1.39052	1.77513	1.19226	1.07173	-0.19589	0.46632
-1.96539	-2.69170	2.12163	0.04801	2.30009	0.50726
-1.28385	0.44869	1.29553	-0.38302	-0.13599	0.54820
